# MSMFusion: A Method for Multiscale Dual-Path State Space Modeling for Multimodal Medical Image Fusion

**DOI:** 10.3390/bios16100576

**Published:** 2026-10-13

**Authors:** Liping Yang, Nairui Sun, Tengchao Sun, Yiming Chen, Yao Xiao, Shu Li, Wei Jia, Shaoshuai Luo, Wei Wang

**Affiliations:** 1 College of Computer Science and Technology, Changchun University, No. 6543, Satellite Road, Changchun 130022, China; yanglp@ccu.edu.cn (L.Y.); 241501517@mails.ccu.edu.cn (N.S.); 241503550@mails.ccu.edu.cn (T.S.); 241503545@mails.ccu.edu.cn (Y.X.); 241501501@mails.ccu.edu.cn (S.L.); 251501560@mails.ccu.edu.cn (W.J.); 251501543@mails.ccu.edu.cn (S.L.); 2 Bethune First Clinical Medical College, Jilin University, Changchun 130021, China; chenym9924@mails.jlu.edu.cn

**Keywords:** multimodal medical image fusion, state space model, multiscale features, structural–functional complementarity

## Abstract

Multimodal medical image fusion aims to integrate information from different imaging techniques to generate a single fused image that is comprehensive and rich in detail. Structural and functional imaging provide high-resolution anatomical details and tissue metabolic information, respectively. Complementary fusion improves quantitative fusion quality and preserves structural and functional information in one view, which aids clinical interpretation. Currently, convolutional neural network-based methods struggle to efficiently capture global features, whereas Transformer-based models excel at global modeling but incur relatively high computational costs. To address the above issues, we propose MSMFusion, a multiscale state-space modeling method for complementary structural–functional medical image fusion. A multiscale feature extraction module first extracts modality-specific multiscale structural features; subsequently, a dual-path MSMamba establishes long-range dependencies with linear complexity: the full-resolution scanning path preserves fine boundaries and textures, while the downsampled scanning path models global context with a more compact sequence; finally, a gated adaptive fusion module estimates spatial–channel weights based on local content to selectively integrate anatomical structure and functional metabolic information. On the Harvard medical image dataset, MSMFusion achieves a mutual information of 2.2181, a peak signal-to-noise ratio of 32.9390, and a structural similarity of 0.7616 on the MRI–PET task, and also outperforms various representative methods based on CNN, GAN, Transformer, and Mamba on the MRI–CT and MRI–SPECT tasks. Compared with the Transformer backbone, MSMamba reduces the number of parameters from 7.201M to 4.154M and the FLOPs from 476.738G to 159.239G, achieving superior fusion quality at lower computational cost while simultaneously preserving fine anatomical structures and key functional information.

## 1. Introduction

Clinical image interpretation often requires grasping both anatomical morphology and physiological metabolism simultaneously. Magnetic resonance imaging (MRI) offers high spatial resolution and clear soft-tissue contrast, making it well suited to delineating anatomical boundaries, whereas positron emission tomography (PET) and single-photon emission computed tomography (SPECT) reflect tissue metabolic activity and functional status [1]. Although the two types of information are spatially complementary, they arise from different imaging mechanisms, and when viewed as separate images, anatomical structure and metabolic activity are difficult to inspect together within a single field of view. Compressing multimodal images into a single, more readable result has therefore become an important topic in medical image analysis [2,3]. For structural–functional fusion, the key lies in stably transferring the metabolic responses of PET and SPECT while preserving the fine anatomical details of MRI, so that structural and functional information can be inspected together in one view. This aids clinical interpretation.

To obtain cross-modal complementary representations, early works mostly relied on hand-crafted transforms and predefined rules [4,5], such as multiscale decomposition, sparse coding, and optimization-based solving [6,7,8]. Although these pipelines are relatively interpretable, their adaptability to complex lesion morphology and modality differences is limited. Deep learning was subsequently introduced into this task, learning features and fusion mappings in a data-driven manner, which improved overall quality [9]. Even so, existing networks still exhibit three notable shortcomings. First, many methods concatenate the source images along the channel dimension and feed them into a single convolutional backbone [10], resulting in insufficient multiscale modeling, whereby fine anatomical structures and local lesion responses are prone to being smoothed out. Second, the limited receptive field of convolutions gives rise to inadequate characterization of long-range dependencies across regions, making global context difficult to align before fusion. Third, cross-modal aggregation is still commonly implemented as feature addition or concatenation, lacking a mechanism for assigning modality weights according to spatial content, so that anatomical textures and functional pseudo-color maps tend to suppress each other. Although Transformers can compensate for long-range modeling, their self-attention has quadratic complexity and incurs prohibitive overhead on high-resolution medical images. State space models offer a linear-complexity path for long-sequence modeling; however, if applied directly, they still lack multiscale representations and explicit cross-modal interactions tailored to medical fusion.

To address the above problems, we propose MSMFusion, which performs complementary structural–functional medical image fusion through multiscale state space modeling. The two source images first enter a parallel multiscale feature extraction module (MSFEM), yielding modality-specific, scale-aligned local representations. Subsequently, a dual-path MSMamba establishes long-range spatial dependencies via structured state space scanning: the full-resolution path preserves boundaries and textures, while the downsampled path aggregates global context over shorter sequences. Finally, a gated adaptive fusion module (GAFM) estimates spatial–channel weights according to local content, selectively combining the anatomical branch and the functional branch to reconstruct a fused image that simultaneously contains soft-tissue details and metabolic information. The proposed method not only effectively captures salient features across local multiscales, but also fully exploits global contextual information, integrating the complementary information across modalities well and further improving fusion performance.

The main contributions are as follows:

(1) We propose an MSMFusion framework for complementary structural–functional fusion, which combines convolutional local modeling with state space long-range modeling to generate fused results with more complete information.
(2) We design a multiscale feature extraction module (MSFEM) to enhance multiscale structural modeling, considering that anatomical structures in magnetic resonance imaging (MRI) images are of relatively small feature sizes. This module obtains receptive fields at different scales through dilated convolutions, and uses channel and spatial attention to guide the network to focus on the structural information unique to each modality, thereby improving the feature extraction capability of the model.
(3) We innovatively design an MSMamba module employing a dual-path scanning expansion strategy, which combines two full-resolution and two downsampled scanning paths. This design achieves comprehensive spatial coverage: the full-resolution branch retains fine-grained details for accurate feature capture, while the downsampled branch generates compact feature sequences, improving the efficiency of long-range modeling without sacrificing contextual richness. In addition, we design an adaptive gated fusion module (GAFM) that adaptively adjusts the contributions of the two modalities according to spatial position, compensating for the lack of explicit cross-modal interactions in state space models.
(4) On the MRI–CT, MRI–PET, and MRI–SPECT tasks of the public Harvard medical imaging dataset, we compare our method with various CNN, GAN, Transformer, and Mamba approaches. MI, VIF, PSNR, and SSIM are the highest on these tasks. EN is lower on all three, and SF is the highest only on MRI–CT. The qualitative comparisons are consistent with these metric differences. The same model is also applicable to visible–infrared fusion. On MSRS and RoadScene, it fuses a visible image with an infrared image of road scenes. PSNR and VIF are the highest on both sets. Entropy is the highest on MSRS and is not on RoadScene, and spatial frequency is lower than several comparison methods.

The remainder of this paper is organized as follows. Section 2 reviews conventional methods for multimodal medical image fusion and the development of state space models. Section 3 details the network architecture and module design of the proposed MSMFusion. Section 4 provides the experimental setup and comparative results. Section 5 analyzes the loss-function weights. Section 6 states the limitations and future work, and Section 7 concludes the paper.

## 2. Related Work

### 2.1. Multimodal Medical Image Fusion Methods

In recent years, deep learning has achieved remarkable progress in the field of multimodal image fusion (MMIF), and researchers have proposed various fusion methods based on different network architectures. Early methods mainly relied on convolutional neural networks and autoencoders, with a typical pipeline of extracting features using CNNs and reconstructing the fused image through a decoder. For example, DenseFuse improves feature propagation efficiency through densely connected structures [11], while NestFuse [12] and RFN-Nest [13] introduce nested structures and multiscale mechanisms to enhance feature representation capability. However, owing to the local receptive field of CNNs, they struggle to model long-range dependencies, which limits their ability to extract global information and consequently affects fusion quality. In addition, the design of shared or independent encoders also faces a trade-off between modality-specific and shared features. To overcome these limitations, researchers introduced generative models, such as the generative adversarial network FusionGAN [14] and diffusion models [15]. FusionGAN is the first work to apply GANs to image fusion, and subsequent methods such as GANMcC [16], DDcGAN [17], and TCGAN [18] further optimized the application of GANs in image fusion. Although generative models can produce fused images of high visual quality [19], issues such as unstable training, high computational complexity, and mode collapse still limit their practicality [20].

CNN-based feature extraction and hybrid feature integration have also been studied for single-modality medical image analysis, and they provide context for the multiscale extractor and the cross-modal fusion rule used in this work. They process optical coherence tomography images with CNNs, keep the dominant activations, and apply the slime mold algorithm to select the activations used for retinal-disease classification [21]. SpiCoNET is a hybrid model for chest X-ray interpretation: convolutional backbones are combined with a spiking neural network, the features of the two most accurate backbones are integrated, and a SEFRON spiking layer classifies the combined representation [22]. These studies show that a convolutional front end followed by an explicit integration step can strengthen medical image representation. The integration in [21] selects activations within one optical coherence tomography image. The integration in [22] combines two convolutional streams of the same radiograph and then classifies them. MSFEM carries the convolutional multiscale idea into paired structural–functional fusion by building parallel dilated receptive fields for each modality, and GAFM then assigns spatial–channel weights after the two modality streams have been modeled separately. MSFusion shows that a fusion rule applied only at the deepest layer discards shallow texture, so this weighting is applied across scales rather than only at the deepest layer [23].

With the increasing demand for global modeling capability, Transformer [24] and its variant Swin Transformer [25] have been gradually introduced into MMIF tasks. Owing to its self-attention mechanism, the Transformer can effectively capture long-range dependencies in images, thereby improving fusion performance. The proposed FATFusion combines spatial and channel attention mechanisms [26] and achieves favorable performance in medical image fusion tasks. However, a pure Transformer architecture is insufficient in modeling local details; therefore, many studies have begun to explore strategies that combine CNNs with Transformers. For example, a dual-branch CNN–Transformer network architecture has been designed, combining the local perception capability of CNNs with the global modeling capability of Transformers [27]; Swin Transformer has also been combined with CNNs for feature extraction [28]; MATR [29] and MACTFusion [30] respectively propose multiscale and cross-modal Transformer modules to enhance inter-modal information interaction. Although these hybrid architectures have achieved significant improvements in fusion performance, the quadratic time complexity and high computational cost brought by the self-attention mechanism remain major challenges that constrain their real-time applications. In summary, existing methods have already made substantial breakthroughs in fusion quality and feature representation, yet there is still a need to further explore efficient and effective backbone architectures to meet the dual requirements of real-time performance and robustness in MMIF tasks.

### 2.2. State Space Models

State space models (SSMs) have attracted considerable attention owing to their superior performance in long-sequence modeling. Through selective scanning mechanisms, they enable long-range dependency modeling of input features, and thanks to their linear computational complexity and parallelism, they have achieved remarkable results in natural language processing and speech recognition, establishing themselves as an important component of deep learning. The structured state space sequence model (S4) is a pioneering work in this field [31], which effectively resolves the difficulty of parallel computation in traditional SSMs by introducing a parameterization based on a diagonalized low-rank structure, thereby enabling efficient modeling of long-range dependencies. Building on this, the S5 layer further proposes a parallel scanning strategy for diagonalized linear SSMs, improving model efficiency [32]; the H3 model extends the capability of S4 and attains performance comparable to that of Transformers in language modeling tasks [33]. Mamba introduces a selective state space mechanism on top of S4 [34], allowing the model to dynamically filter key information according to the input, and combines this with a hardware-aware algorithm to substantially improve training and inference efficiency. Compared with Transformers of comparable scale, Mamba demonstrates higher throughput, faster inference speed, and superior overall performance. Following its success in natural language processing (NLP) tasks, Mamba has been gradually extended to computer vision, such as Vision Mamba (Vim) [35] and Visual State Space Models (VMamba) [36], which enhance image representation capability through bidirectional Mamba blocks and cross-scan mechanisms, respectively.

Inspired by the success of Mamba in unimodal visual recognition, selective state space models have been extended to cross-modal tasks. FMamba applies Mamba to image dehazing [37], and a cross-modal Mamba framework has been developed for object detection [38]. For image fusion, MambaDFuse [39], FusionMamba [40], and SSEFusion [41] show that linear-complexity scanning can replace quadratic self-attention. These fusion models differ in scan resolution, multiscale construction, and cross-modal weighting.

### 2.3. Comparison with Mamba-Based Image Fusion Methods

VMamba [36], MambaDFuse [39], FusionMamba [40], SSEFusion [41], and MSMFusion are compared on scanning strategy, multiscale representation, and cross-modal weighting. FMamba [37] and the detection model [38] are omitted because neither produces a fused image.

Scanning strategy. VMamba scans four directions at the original resolution, so additional directions repeat tokens of the same length [36]. MambaDFuse uses a single-resolution selective scan and does not separate a detail path from a context path [39]. FusionMamba skip-samples every direction and then restores neighborhood relations with a local convolution, so boundary locations are not kept as individual tokens during the state update [40]. SSEFusion uses Mamba for long-range medical semantics together with dynamic spiking neural networks, without changing the scan resolution [41]. MSMamba uses two full-resolution paths and two stride-2 paths, so detail retention and long-range aggregation are carried by sequences of different lengths.

Multiscale modeling. MambaDFuse obtains scale by stacking shallow convolutions and deep Mamba blocks, which gives two feature levels rather than parallel receptive fields [39]. FusionMamba changes scale by patch merging across U-Net stages [40]. SSEFusion emphasizes salient semantic responses [41]. MSFEM builds parallel dilated receptive fields, together with global pooling and channel–spatial attention, before the features enter MSMamba.

Adaptive fusion. MambaDFuse exchanges channels with a fixed mask and then uses the two modality streams to guide a fused sequence inside its multi-modal Mamba block [39]. FusionMamba mixes the enhanced modality features inside a cross-modal Mamba module [40]. SSEFusion steers the fused representation with dynamic spiking neurons [41]. GAFM leaves the two modality streams unmixed through feature extraction and scanning, and then predicts spatial–channel weights from their joint local representation.

MambaDFuse, FusionMamba, and SSEFusion place their emphasis on different parts of this problem: single-resolution or skip-sampled scanning, scale obtained from network depth, and cross-modal mixing inside the state update. MSMFusion combines dilated multiscale features, a dual-resolution scan, and a spatial–channel gate applied after the two modalities have been modeled independently. These differences are summarized in Table 1.

## 3. Method

### 3.1. Overall Architecture

**Figure 1:**
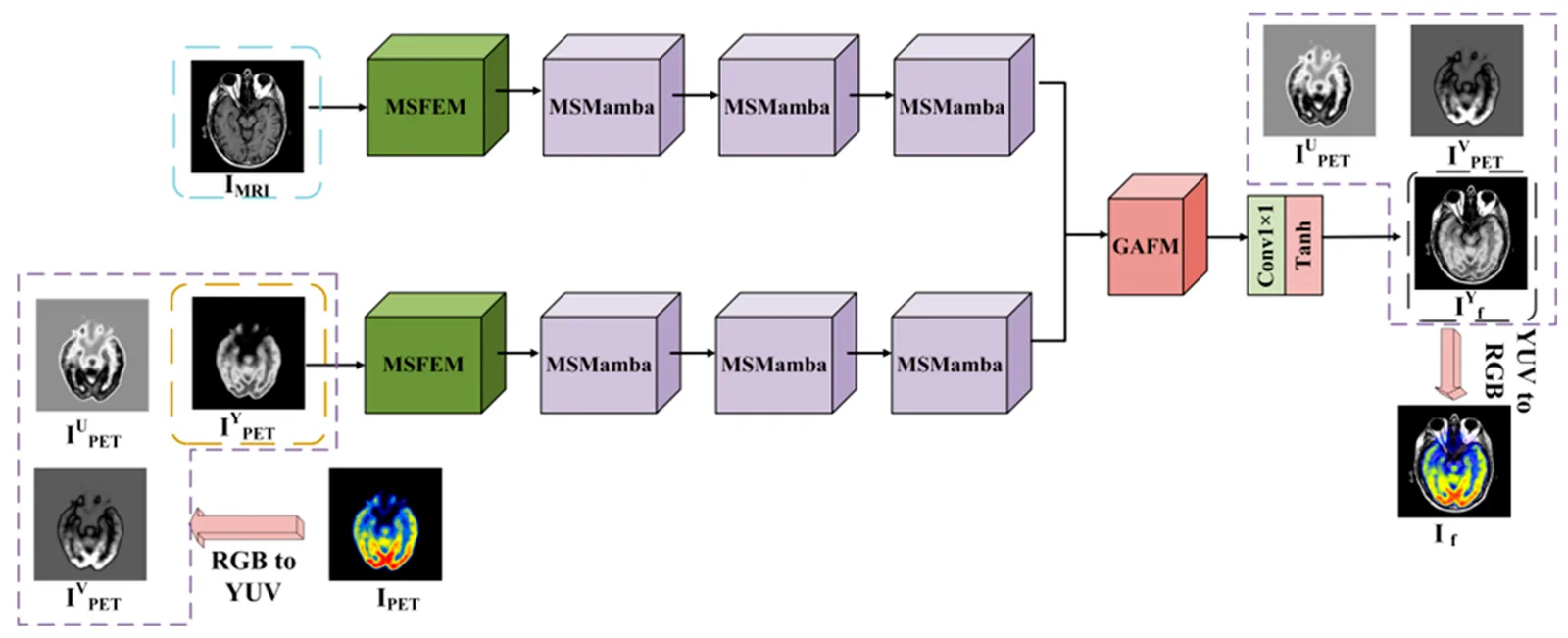
Overall framework of the proposed MSMFusion method.

Given the pronounced feature differences between MRI and PET images, it is essential to preserve important complementary information while suppressing redundant features. To this end, we propose a multimodal medical image fusion method, MSMFusion. The overall architecture is illustrated in Figure 1. Considering that the PET image *I*_PET_ is an RGB image whereas the MRI image *I*_MRI_ is a single-channel grayscale image, we adopt an RGB-to-YUV color transformation to resolve the channel mismatch. RGB–YUV conversion is widely used to fuse three-channel functional biomedical images with single-channel structural images because it effectively separates luminance from chrominance and thereby facilitates more meaningful cross-modal feature integration. Specifically, in the initial stage, *I*_PET_ is converted into the YUV color space, yielding a luminance component $I_{Y}^{\mathrm{PET}}$ and two chrominance components $I_{U}^{\mathrm{PET}}$ and $I_{V}^{\mathrm{PET}}$. The luminance component $I_{Y}^{\mathrm{PET}}$ and the MRI image *I*_MRI_ are then fed into the MSFEM to model multiscale information for each modality. Next, the extracted multiscale features are processed by the multiscale Mamba module (MSMamba) to establish long-range dependencies and global contextual representations. Finally, a fusion strategy based on the GAFM promotes feature interaction and fusion, producing a fused image that contains complementary information from both modalities. After fusion, the fused luminance component $I_{Y}^{f}$ is combined with the original chrominance components $I_{U}^{\mathrm{PET}}$ and $I_{V}^{\mathrm{PET}}$ and converted back to the RGB color space via a YUV-to-RGB transformation, yielding the fused result *I_f_*.

### 3.2. Multiscale Feature Extraction Module

**Figure 2:**
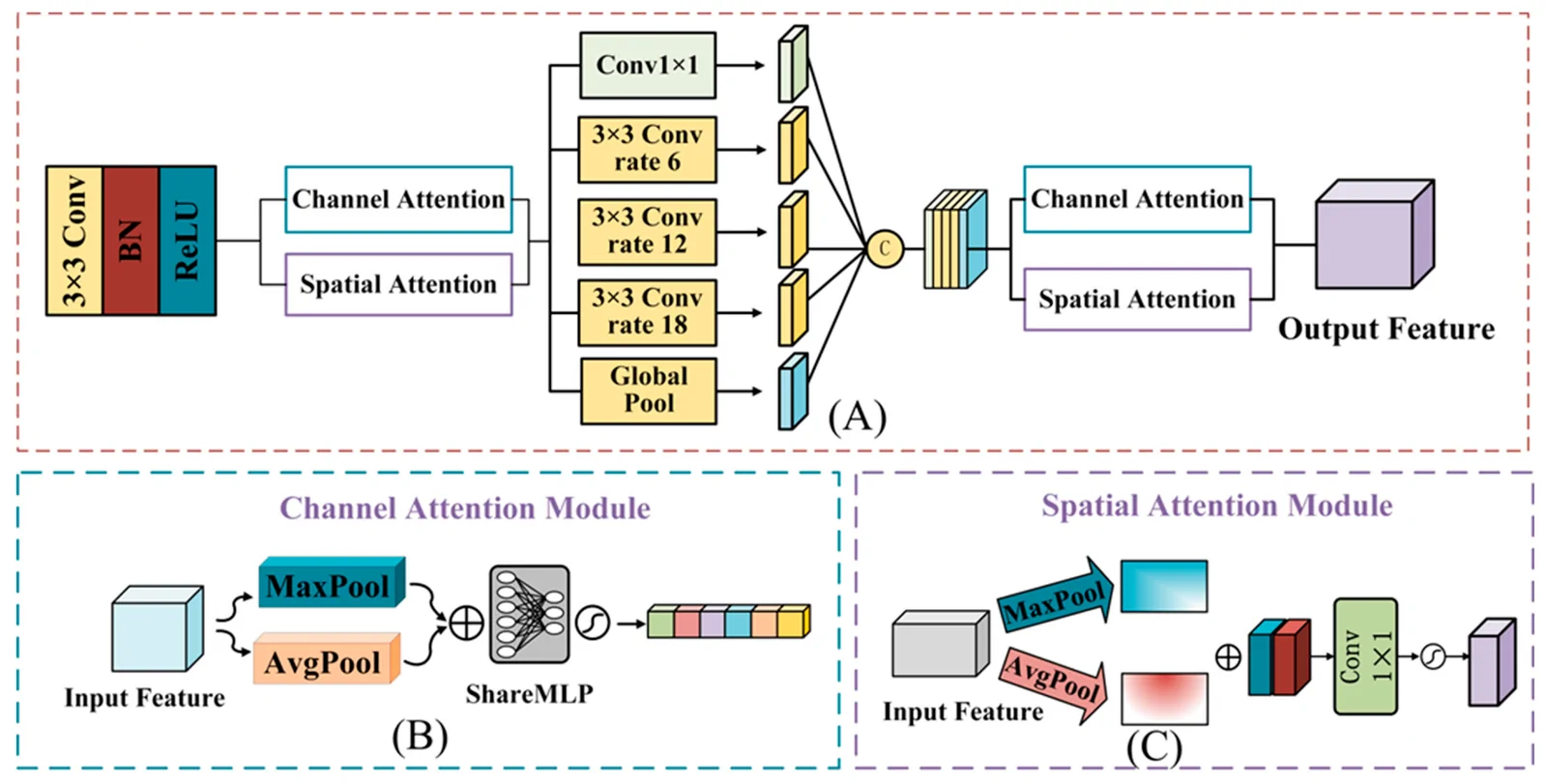
Architecture of the multiscale convolutional MSFEM module.

Lesions and anatomical structures in medical images often span multiple spatial scales; if shallow features are fed directly into state-space scanning, fine-structure responses are easily diluted by global sequence modeling. LPM-Net shows that a convolutional front end and state-space scanning can be combined for medical image fusion [42]. To this end, we design an MSFEM module that fully exploits multiscale complementary information from different modalities and provides the subsequent MSMamba module with scale-aligned, more modality-discriminative input representations, as illustrated in Figure 2. Shallow features are first extracted by a 3 × 3 convolution and then enhanced by channel attention and spatial attention to strengthen the modality-specific structural information [43]. Concretely, channel dependencies are captured by global average pooling (GAP) and global max pooling (GMP). The resulting feature descriptors are then passed through a shared multilayer perceptron (MLP) to generate adaptive channel attention weights. This enables the network to learn the relative importance of each channel automatically, highlighting informative channels and suppressing redundant responses. The process is formulated as follows:

(1) $$\begin{array}{c} x_{1}=\mathrm{ReLU}(\mathrm{BN}({\mathrm{Conv}}_{3\times 3}(x))) \end{array}$$

(2) $$\begin{array}{c} \mathrm{AvgPool}\left(x_{1}\right)=\frac{1}{H\cdot W}\sum_{i=1}^{H}\sum_{j=1}^{W}x_{c,i,j} \end{array}$$

(3) MaxPoolx1=Hmax i=1Wmaxj=1xc,i,j

(4) $$\begin{array}{c} M_{c}\left(x_{1}\right)=\sigma \left({\mathrm{Conv}}_{1\times 1}\left(\mathrm{ReLU}\left({\mathrm{Conv}}_{1\times 1}\left(\mathrm{AvgPool}\left(x_{1}\right)+\mathrm{MaxPool}\left(x_{1}\right)\right)\right)\right)\right) \end{array}$$

where *x_c_*_,_*_i_*_,_*_j_* denotes the value of the input feature map x at channel c, height i, and width j; *σ* denotes the sigmoid function; and *M_c_*(*x*) is the channel attention weight with shape *C* × 1 × 1. Compared with standard channel attention, this formulation reduces redundant MLP computation by fusing the GAP and GMP descriptors before they are fed into the MLP. Consequently, the MLP learns a joint representation of GAP and GMP rather than modeling them separately and then combining the results. This design helps capture the interaction between GAP and GMP, enhances feature interaction, and improves generalization. The spatial attention module subsequently captures spatial dependencies via GAP and GMP along the channel dimension and generates a spatial attention map through convolution, thereby reducing attention to irrelevant regions and producing the refined feature map. This process is formulated as follows:

(5) $$\begin{array}{c} \mathrm{AvgPool}\left(x_{1}\right)=\frac{1}{C}\sum_{c=1}^{C}x_{c,i,j} \end{array}$$

(6) MaxPoolx1=Cmax c=1xc,i,j

(7) $$\begin{array}{c} M_{s}\left(x_{1}\right)=\sigma \left(\mathrm{Conv}\left(\mathrm{Concat}\left(\left[\mathrm{AvgPool}\left(x_{1}\right),\mathrm{MaxPool}\left(x_{1}\right)\right]\right)\right)\right) \end{array}$$

(8) $$\begin{array}{c} x_{2}=M_{s}\left(M_{c}\left(x_{1}\right)\cdot x_{1}\right)\cdot \left(M_{c}\left(x_{1}\right)\cdot x_{1}\right) \end{array}$$

where *σ* denotes the sigmoid function, *M_s_*(*x*) is the spatial attention weight with shape 1 × *H* × *W*, and *x*_2_ is the enhanced feature map. The features are then fed into a five-branch module. One branch contains a 1 × 1 convolution to further extract low-level features. Three additional branches each contain a 3 × 3 convolution with a different dilation rate (6, 12, and 18, respectively) [44], which is used to capture contextual information of medical images at different scales. The remaining branch employs a global average pooling layer to obtain global image features. This process is formulated as follows:

(9) $$\begin{array}{c} x_{3}=\mathrm{ReLU}\left(\mathrm{BN}\left({\mathrm{Conv}}_{1\times 1}\left(x_{2}\right)\right)\right) \end{array}$$

(10) $$\begin{array}{c} x_{k}=\mathrm{ReLU}\left(\mathrm{BN}\left({\mathrm{Conv}}_{3\times 3,d=k}\left(x_{2}\right)\right)\right)k\in \left\{6,12,18\right\} \end{array}$$

(11) $$\begin{array}{c} g_{p}=\mathrm{ReLU}\left(\mathrm{BN}\left({\mathrm{Conv}}_{1\times 1}\left(\mathrm{GlobalAvgPool}\left(x_{2}\right)\right)\right)\right) \end{array}$$

where k denotes the dilation rate. The feature maps are upsampled by bilinear interpolation so that they share the same spatial size. The outputs of the five branches are then concatenated and passed through a 1 × 1 convolution for dimensionality reduction, which lowers the model complexity. This process is formulated as follows:

(12) $$\begin{array}{c} g_{p}^{\prime }=\mathrm{Interpolate}\left(g_{p},\mathrm{size}=\left(H,W\right)\right),k\in \left\{6,12,18\right\} \end{array}$$

(13) $$\begin{array}{c} z=\mathrm{Concat}\left(\left[x_{3},x_{k}\ldots ,g_{p}^{\prime }\right]\right) \end{array}$$

(14) $$\begin{array}{c} y=\mathrm{ReLU}\left({\mathrm{Conv}}_{1\times 1}\left(z\right)\right) \end{array}$$

The output feature map y is again fed into the channel attention and spatial attention modules to strengthen the distinctive feature information of each modality. This process is formulated as follows:

(15) $$\begin{array}{c} Y=M_{s}\left(M_{c}\left(y\right)\cdot y\right)\cdot \left(M_{c}\left(y\right)\cdot y\right) \end{array}$$

where Y denotes the final feature map.

### 3.3. MSMamba Module

**Figure 3:**
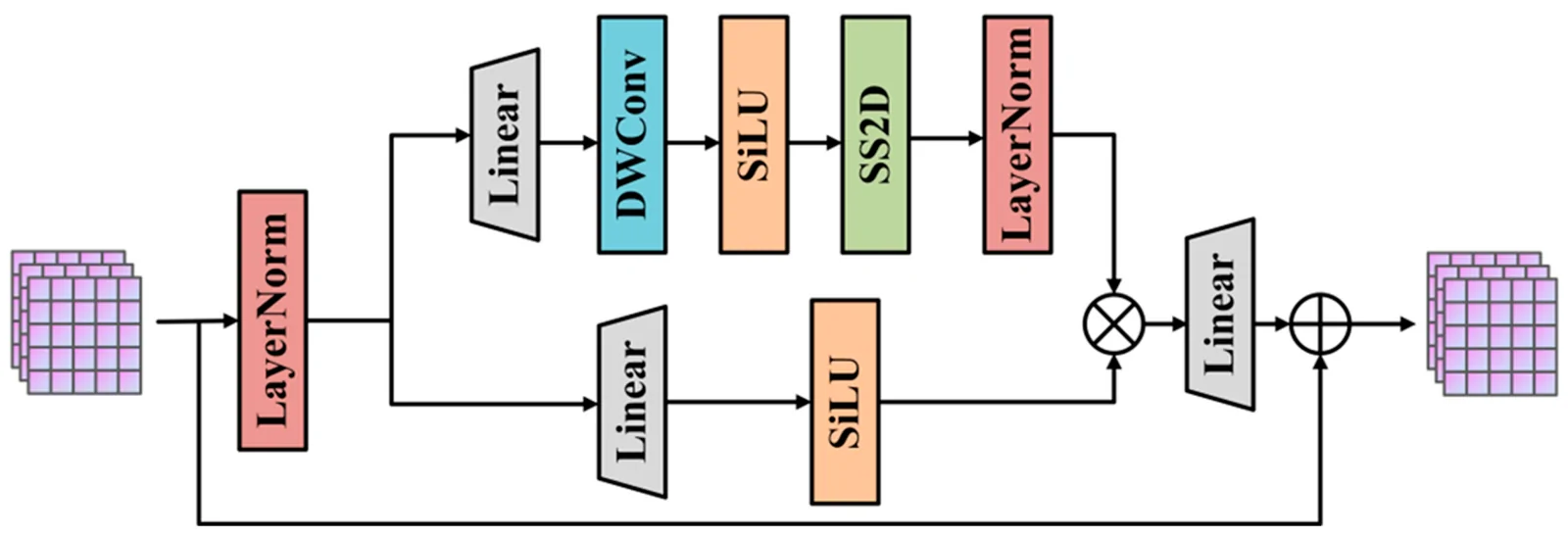
Architecture of the proposed MSMamba.

Standard visual state space models mostly employ multi-directional scanning to enhance spatial coverage. However, in high-resolution medical images, full-resolution multi-path scanning often introduces significant feature redundancy and sequence overhead; conversely, relying solely on downsampled sequences tends to lose the detailed information necessary for boundary localization. Therefore, after extracting multiscale features from different modalities, it is essential to effectively capture global and local contextual information and establish long-range feature dependencies. To this end, we design an MSMamba module. As shown in Figure 3, the input features **X** ∈ ℝ***^H^***^×^***^W^***^×^***^C^*** are first fed into two parallel branches. In the first branch, the features are processed sequentially by a linear layer (Linear), a depthwise separable convolution (DWConv) [45], a sigmoid linear unit (SiLU) activation function, an SS2D layer, and an LN layer. In the second branch, the features pass through a linear layer (Linear) and a SiLU activation function. The features from the two branches are merged via element-wise multiplication, and then aggregated with the input features through a linear layer to output the final feature map. This process is expressed as follows:

(16) $$\begin{array}{c} F_{1}=\mathrm{LN}\left(\mathrm{SS}2D\left(\sigma \left(\mathrm{DWConv}\left(\mathrm{Linear}\left(Y\right)\right)\right)\right)\right) \end{array}$$

(17) $$\begin{array}{c} F_{2}=\sigma \left(\mathrm{Linear}\left(Y\right)\right) \end{array}$$

(18) $$\begin{array}{c} F_{\mathrm{out}}=Y+\mathrm{Linear}\left(F_{1}\left(\mathrm{LN}\left(x\right)\right)⨂F_{2}\left(\mathrm{LN}\left(x\right)\right)\right) \end{array}$$

where LN denotes layer normalization, *σ* denotes the SiLU function, and ⊗ denotes element-wise multiplication.

**Figure 4:**
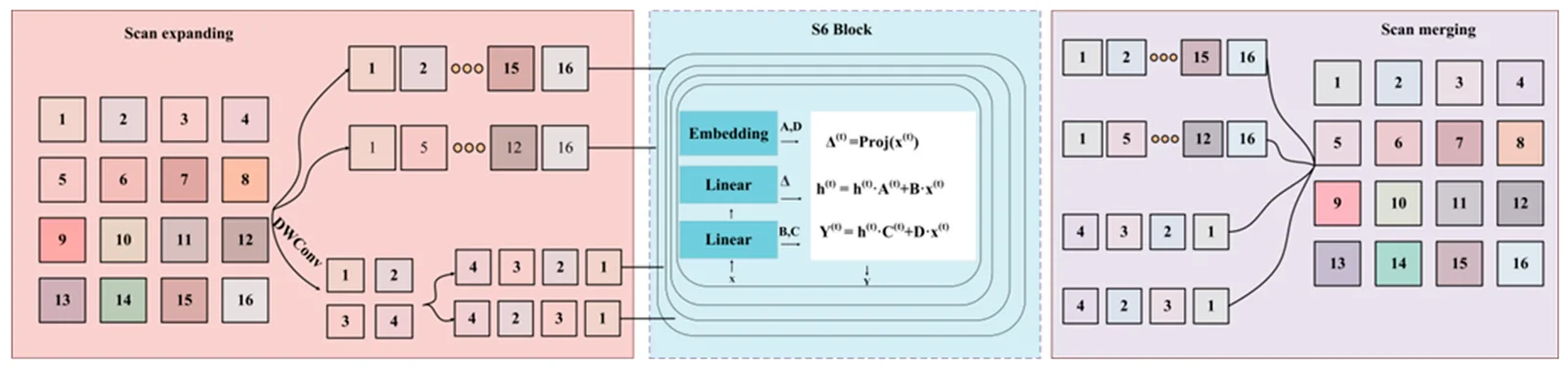
Visualization of the internal modeling process of SS2D.

SS2D consists of three components: a scan expansion operation, an S6 block, and a scan merging operation [36], as illustrated in Figure 4. The scan expansion operation generates multiscale features from the input image through DWConv with different strides, and then unfolds them into sequences along four different directions. These four scanning paths are divided into two groups: two of them maintain the original resolution and are processed by the S6 block to extract features, ensuring that information from each direction is comprehensively scanned and that diverse features are captured; the other two are downsampled via DWConv with a stride of 2 before scanning. After being processed by the S6 block, they are upsampled through bilinear interpolation. This strategy enables long-range modeling to be completed on shorter sequences, reducing the sequence and feature redundancy introduced by repeated full-resolution scanning. Subsequently, the scan merging operation sums and merges the sequences from the four directions, restoring the output image to the same size as the source image. This process is expressed as follows:

(19) $$\begin{array}{c} y_{1},y_{2}=\mathrm{SSM}\left(\mathrm{Reshape}\left({\mathrm{Dwconv}}_{\mathrm{stride}=i}\left(x\right),\left[B,C,H\cdot W\right]\right)\right) \end{array}$$

(20) y3,y4=SSMReshapeTranspose(Dwconvstride=i(x),dim=2,dim=3),B,C,H·Wi∈1,2

(21) $$\begin{array}{c} y_{i}=\mathrm{LN}\left(\mathrm{Reshape}\left(y_{i=1,2,3,4},\left[B,C,H,W\right]\right)\right) \end{array}$$

(22) $$\begin{array}{c} Y_{\mathrm{out}}=y_{1}+y_{2}+\mathrm{Interpolate}\left(y_{3}+y_{4}\right) \end{array}$$

where Reshape denotes a shape transformation (e.g., from (*B*, *C*, *H*, *W*) to (*B*, *C*, *H* · *W*)) that flattens the spatial dimensions, Transpose denotes the transpose operation, and Interpolate denotes bilinear interpolation.

### 3.4. Gated Adaptive Fusion Module

**Figure 5:**
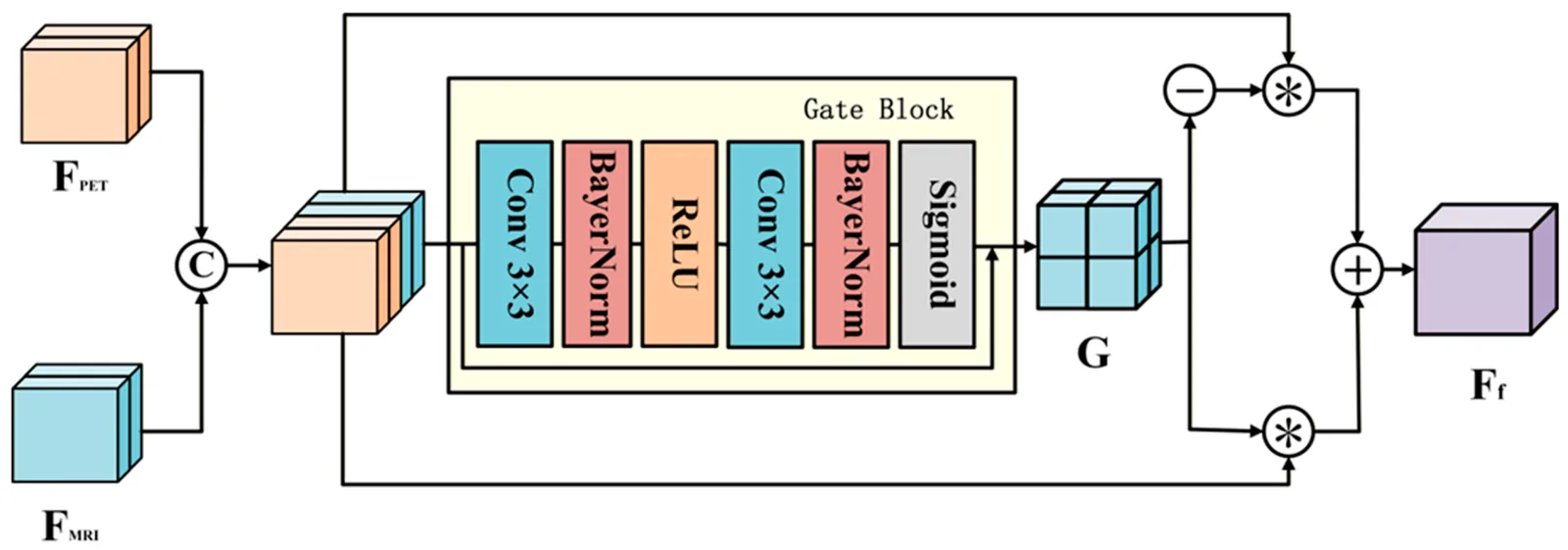
Architecture of the gated adaptive fusion module.

After MSMamba completes global context modeling for each modality, it remains necessary to effectively integrate the representations of the anatomical branch and the functional branch. However, most conventional fusion strategies are based on weighted averaging or max-fusion methods. Although these traditional methods are simple and effective, the core problem is that the design of the weight map is often insufficiently flexible; particularly when processing deep features, they cannot identify the importance of each modality, which easily leads to information loss or semantic ambiguity. To address these issues, we design a gated adaptive fusion module (GAFM), which compensates for the lack of explicit cross-modal interactions in state space models, as illustrated in Figure 5. GAFM adaptively estimates modality importance at each spatial position according to local content, thereby performing content-conditioned fusion of the two feature streams. Let the output features of the PET and MRI branches be as follows:

(23) $$\begin{array}{c} F^{\mathrm{PET}},F^{\mathrm{MRI}}\in R^{C\times H\times W} \end{array}$$

where C is the number of channels and H and W are the spatial dimensions of the feature maps. First, the features of the two modalities are concatenated along the channel dimension to obtain a joint representation:

(24) $$\begin{array}{c} F_{c}=\mathrm{Concat}\left(F^{\mathrm{PET}},F^{\mathrm{MRI}}\right)\in R^{2C\times H\times W} \end{array}$$

The joint feature is passed through a sub-network consisting of two convolutional layers with batch normalization and an intermediate ReLU activation, forming a nonlinear mapping. An element-wise sigmoid function then compresses the output to the interval (0, 1), yielding a joint spatial–channel gating map:

(25) $$\begin{array}{c} G=\sigma \left(\mathrm{BN}\left({\mathrm{Conv}}_{3\times 3}\left(\mathrm{ReLU}\left(\mathrm{BN}\left({\mathrm{Conv}}_{3\times 3}\left(F_{c}\right)\right)\right)\right)\right)\right), \end{array}$$

where *G_c_*_,_*_i_*_,_*_j_* denotes the weight of the MRI modality at channel c and spatial location (*i*, *j*), and 1 − *G_c_*_,_*_i_*_,_*_j_* denotes the corresponding weight of the PET modality. The fused feature is finally obtained by element-wise weighted summation of the two modality features:

(26) $$\begin{array}{c} F_{f}=G\cdot F^{\mathrm{MRI}}+\left(1-G\right)\cdot F^{\mathrm{PET}} \end{array}$$

where *F_f_* is the fused feature. Complementary information from the two modalities is thus adaptively regulated at every channel and spatial location. This mechanism enables the model to preserve the functional information in PET images that reflects tissue metabolic activity, while fully retaining the high-resolution anatomical structure and soft-tissue contrast provided by MRI.

## 4. Experiments

### 4.1. Experimental Settings

**Datasets.** In our study, the datasets were downloaded from a mainstream medical image database, namely the Harvard Medical Database [46]. Three datasets were used to validate our fusion model. Specifically, the training set consists of 184 pairs of MRI–CT images, 269 pairs of MRI–PET images, and 357 pairs of MRI–SPECT images. In addition, 21 pairs of MRI–CT images, 42 pairs of MRI–PET images, and 73 pairs of MRI–SPECT images were selected as the test set to comprehensively validate the fusion performance. These images come from the public Harvard Whole Brain Atlas. In that atlas, each clinical case is one patient and contains multiple co-registered transaxial slices. The atlas releases these slices as registered pairs and does not provide a patient-level training–test partition. The pairs were therefore selected by image pair, giving the training and test counts stated above. A patient-level split was not applied. Because one patient contributes multiple slices, and the pairs were not kept together by patient, slices from the same patient can be assigned to both the training set and the test set. The same model is also applied to visible–infrared fusion, and the comparison shows that it remains applicable on this task. MSRS contains paired infrared grayscale images and visible color images of road scenes. This experiment follows the official split, with 1083 training pairs and 361 test pairs. Of the training pairs, 536 were acquired in daytime and 547 at night; of the test pairs, 179 were acquired in daytime and 182 at night [47]. RoadScene contains 221 registered pairs of infrared grayscale images and visible color images. The scenes include roads, vehicles, and pedestrians. Using random seed 2026, the experiment assigns 177 pairs to training and 44 pairs to testing [48]. The modality combination and the scene content differ from the MRI–CT, MRI–PET, and MRI–SPECT pairs.

**Implementation details.** The experiments were conducted on an NVIDIA GeForce RTX 4090 GPU and implemented using the PyTorch framework (version 2.1.2). During training, the Adam optimizer [49] was employed, with an initial learning rate set to 1 × 10^−3^, a batch size of four, and the number of training epochs fixed at 100. For the CT–MRI fusion task, both source images are grayscale, so no color space conversion is required and they can be directly fed into the network. For the PET–MRI and SPECT–MRI tasks, the PET and SPECT functional images are in RGB format. To eliminate the channel mismatch with the grayscale MRI, we follow the common practice in medical image fusion by first mapping them to the YUV color space, thereby decoupling luminance and chrominance information. Specifically, the luminance component Y, which carries the main anatomical–functional structure and intensity information, is fed into the fusion network together with the grayscale MRI image for training and inference, while the chrominance components U and V are cached separately and kept strictly spatially aligned with the corresponding Y, without participating in network training. After the network outputs the fused luminance component *Y_f_*, it is concatenated with the original unmodified U and V components, and the final RGB fused image is obtained through the inverse YUV-to-RGB transformation. This strategy decouples cross-modal structural fusion from color information, preserving the original pseudo-color appearance of the functional images while avoiding unnecessary mixing of chrominance channels that carry no cross-modal structural significance. All the experiments employed the same configuration. Each modality stream contains one MSFEM and three MSMamba blocks, the depth retained after comparing stacks of one, two, three, and four blocks. Each block uses two full-resolution scanning paths and two stride-2 scanning paths, and the two streams are combined by the GAFM. MRI–CT, MRI–PET, and MRI–SPECT use the same optimizer, learning rate, batch size, and number of epochs. The loss weights are given below.

**Loss Function.** To obtain high-quality fused results in multimodal medical image fusion tasks while preserving the key information and visual details in the source images, we construct the loss function from four aspects: intensity loss, gradient loss, structural loss, and total variation regularization loss, which supervise the model training at the pixel level, edge level, and perceptual level, respectively. The specific formulation is as follows:

(27) $$\begin{array}{c} L_{\mathrm{total}}={ℷ}_{1}L_{\mathrm{Inten}}+{ℷ}_{2}L_{\mathrm{Grad}}+{ℷ}_{3}L_{\mathrm{SSIM}}+{ℷ}_{4}L_{\mathrm{TV}} \end{array}$$

where ℒ_Inten_, ℒ_Grad_, ℒ_SSIM_, and ℒ_TV_ denote the intensity loss, gradient loss, structural loss, and TV regularization loss, respectively, and *λ*_1_, *λ*_2_, *λ*_3_, and *λ*_4_ are the corresponding fixed weights. They are set to *λ*_1_ = 20, *λ*_2_ = 100, *λ*_3_ = 100, and *λ*_4_ = 1. The numerical magnitudes and task priorities of the four terms differ: the intensity and gradient losses, which are based on pixel-wise absolute differences and the Sobel operator, are typically small in magnitude, whereas the SSIM-based structural loss generally lies in the range [0, 1]. Meanwhile, medical image fusion places particular emphasis on edge sharpness and structural fidelity. Therefore, relatively large weights (100) are assigned to the gradient and structural losses, and a moderate weight (20) is assigned to the intensity loss, so as to balance their gradient contributions during back-propagation and to emphasize structure and texture preservation. The TV weight is set to 1 and serves only as a weak smoothness regularizer, thereby avoiding excessive suppression of details. This setting is consistent with common practice in medical image fusion and was verified in preliminary experiments, yielding the best overall performance.

The intensity consistency loss is used to ensure that the fused image retains the most salient luminance information from the two input modalities. It is formulated as follows:

(28) $$\begin{array}{c} L_{\mathrm{Inten}}={\left\|I_{\mathrm{fused}}-\max(I_{A},I_{B})\right\|}_{1} \end{array}$$

where *I*_fused_ denotes the fused image, ‖ ‖_1_ denotes the $l_{1}$ norm, *I_A_* and *I_B_* denote the MRI and PET (or the corresponding source) images, and *max*(·,·) denotes the element-wise maximum.

Gradient consistency loss. This term extracts gradient maps of the source and fused images with the Sobel operator and uses an L1 loss to make the gradient distribution of the fused image as close as possible to the maximum gradient response of the source images. This mechanism effectively preserves boundary sharpness and helps prevent blurring and artifacts. It is formulated as follows:

(29) $$L_{\mathrm{Gead}}=\|G\left(I_{\mathrm{fused}}\right)-\max(\left|G\left(I_{A}\right)\right|,\left|G\left(I_{B}\right)\right|{\|}_{1}$$

where *G*(·) denotes the Sobel gradient operator and |·| denotes the absolute value.

Structural similarity loss. This term guides the model to preserve key structural characteristics of the source images and to generate fused images that better match the preferences of the human visual system. It is formulated as follows:

(30) $$\begin{array}{c} L_{\mathrm{SSIM}}=1-\omega_{1}\mathrm{SSIM}\left(I_{\mathrm{fused}},I_{A}\right)-\omega_{2}\mathrm{SSIM}\left(I_{\mathrm{fused}},I_{B}\right) \end{array}$$

where SSIM(·) denotes the structural similarity index [50], *I*_fused_ is the fused image, *I_A_* and *I_B_* are the source images, and *ω*_1_ and *ω*_2_ are weighting coefficients.

The total-variation loss serves as a smoothness regularizer. By penalizing abrupt changes between adjacent pixels, it effectively suppresses noise and artifacts and improves the spatial continuity of the fused image. It is formulated as follows:

(31) $$\begin{array}{c} L_{\mathrm{TV}}=\frac{1}{C\cdot H\cdot W}\sum_{i,j}\left({|I}_{i+1,j}-I_{i,j}{|}^{2}+({|I}_{i,j+1}-I_{i,j}{|}^{2}\right) \end{array}$$

where C, H, and W denote the number of channels, height, and width of the image, respectively, and *I_i_*_,_*_j_* denotes the pixel value at location (*i*, *j*).

### 4.2. Comparison Methods and Evaluation Metrics

Ten representative and state-of-the-art methods were adopted for qualitative and quantitative comparison, namely the CNN-based method U2Fusion [48], MMIFINet [51], and SAFusion [52], the GAN-based method DDcGAN [17], the Transformer-based methods SwinFuse [53], SwinFusion [28], MATR [29], and FATFusion [26], and the Mamba-based methods FusionMamba [40] and MambaDFuse [39].

We adopted seven evaluation metrics for comprehensive quantitative assessment, including: entropy (EN), spatial frequency (SF) [54], mutual information (MI) [55], peak signal-to-noise ratio (PSNR), structural similarity (SSIM), visual information fidelity (VIF) [56], and *Q^AB^*^/^*^F^* [57].

Entropy (EN) is used to measure the richness of information contained in the fused image. Spatial frequency (SF) measures the edge and texture information of an image; the larger the value, the clearer the image and the more details it contains. Mutual information (MI) evaluates the interdependence between the input images and the final fused image; the larger this metric, the stronger the ability of the fused image to retain information from the input images, indicating that the fused image preserves the unique features and detailed information of the inputs well. Peak signal-to-noise ratio (PSNR) measures pixel-level fidelity against the two source images, which are the reference images used for this calculation. A higher PSNR indicates closer pixel-level agreement with those reference images. Structural similarity (SSIM) measures the structural preservation capability between images, comprehensively considering three factors—luminance, contrast, and structure—to assess whether the image retains key structural information such as edges and textures. Visual information fidelity (VIF) measures the preservation of image information at multiple scales, i.e., whether the fused image can provide the same visual information as the input images. *Q^AB^*^/^*^F^* is a gradient-based measure of how much edge information from the two source images is retained in the fused image. It does not use a fused ground-truth image.

Applicability of the evaluation metrics. Fusion has no single ground-truth image, so these metrics are not restoration scores against a target fused image. They are used because they describe complementary aspects of the fused result, including information retained from the sources, pixel agreement, structure, visual information, edge transfer, gray-level dispersion, and spatial detail. The reference images for PSNR, SSIM, and VIF are the two source images, denoted A and B. The reported value of each is the mean of the two source-referenced scores. Entropy (EN) and spatial frequency (SF) are computed on the fused image alone. Higher scores indicate higher fusion quality on the corresponding aspect. These metric results aid clinical interpretation.

#### 4.2.1. MRI–CT Evaluation Metrics

Table 2 presents the quantitative comparison results of seven metrics on the MRI–CT dataset (mean ± standard deviation). From the perspective of comparison methods, different networks tend to dominate only on a few metrics, and their overall performance is not balanced. Specifically, MambaDFuse and MATR perform prominently on EN (5.4149 and 5.3666, respectively), indicating that their fused results have high grayscale information entropy; however, their SSIM and *Q^AB^*^/^*^F^* are noticeably low, suggesting that the high information entropy has not been effectively translated into structural fidelity and edge transfer capability. FusionMamba achieves the second-best result on SF (31.6786), showing certain sensitivity to high-frequency details, but its MI, SSIM, and VIF are relatively lagging, reflecting insufficient cross-modal information consistency and visual fidelity. U2Fusion performs well on MI, PSNR, and VIF (2.2613, 32.0592, and 0.7022, respectively), while SwinFusion and FATFusion are competitive in terms of *Q^AB^*^/^*^F^* or SSIM. In contrast, DDcGAN and SwinFuse lag noticeably behind on multiple metrics; in particular, SwinFuse’s SF and *Q^AB^*^/^*^F^* are significantly low, indicating obvious deficiencies in clarity and edge information preservation in its fused results. Overall, existing methods struggle to simultaneously achieve stable advantages across information retention, structural consistency, visual fidelity, and edge transfer.

MSMFusion is highest on six of the seven MRI–CT metrics. SF, MI, PSNR, SSIM, VIF, and *Q^AB^*^/^*^F^* are 32.0921, 2.3306, 32.3425, 0.7267, 0.7166, and 0.5876. The separations are clearest for MI and SF; SSIM exceeds SAFusion (0.7180) by less, and edge preservation exceeds SwinFusion (0.5635) by less. EN is the disadvantage. At 4.9133, it is lower than MMIFINet (5.5939), MambaDFuse, MATR, SwinFusion (4.9518), and FATFusion (4.9414), so the gray-level distribution is less dispersed. Detail activity, information retention, pixel fidelity, and edge transfer are the advantages on this dataset, and entropy is not.

#### 4.2.2. MRI–PET Evaluation Metrics

Table 3 presents the quantitative comparison results of seven metrics on the MRI–PET dataset (mean ± standard deviation). The comparison methods exhibit a characteristic of “single-point prominence with overall imbalance” across different metrics. FATFusion achieves the highest EN (5.0443), but its MI and PSNR are relatively low, indicating that the high information entropy has not been effectively translated into cross-modal information consistency and reconstruction fidelity. FusionMamba performs best on SF (26.9397), showing high sensitivity to spatial detail variations; however, its SSIM drops significantly to 0.3104, suggesting that the fusion process may have introduced over-smoothing or structural distortion, thereby weakening anatomical structural fidelity. MATR and SwinFuse are overall weak on metrics such as MI and VIF, and the collaborative expression of functional and structural information is insufficient. In contrast, SwinFusion and U2Fusion are close to optimal on most metrics, demonstrating more balanced fusion capability; however, they still fail to lead simultaneously on key metrics such as MI, VIF, and *Q^AB^*^/^*^F^*, indicating room for improvement in functional chrominance preservation and edge transfer.

On MRI–PET, MSMFusion achieves the highest MI, VIF, *Q^AB^*^/^*^F^*, PSNR, and SSIM. Its MI is 2.2181, compared with 2.1834 for SAFusion. Its VIF is 0.8208 and its *Q^AB^*^/^*^F^* is 0.6965, above SwinFusion (0.8140) and MambaDFuse (0.6870). Its PSNR and SSIM are 32.9390 and 0.7616, only slightly above SwinFusion (32.9349) and U2Fusion (0.7607). Its EN and SF are the two lower scores: EN is 4.3326, below MMIFINet (5.5390) and FATFusion (5.0443), and SF is 25.3561, below FusionMamba (26.9397), SAFusion (26.6431), and FATFusion (25.6874). For MSMFusion, the advantages lie in retained source information, visual information, transferred edges, pixel agreement, and structure, while the disadvantages lie in gray-level dispersion and spatial frequency.

#### 4.2.3. MRI–SPECT Evaluation Metrics

Table 4 presents the quantitative comparison results of seven metrics on the MRI–SPECT dataset (mean ± standard deviation). From the comparison methods, FATFusion performs best on EN (4.7266), but its PSNR and *Q^AB^*^/^*^F^* are not prominent, indicating that the improvement in information entropy does not stably bring higher fidelity and edge quality. MATR achieves the highest SF (23.6720), showing high sensitivity to spatial frequency variations, but its MI, PSNR, and *Q^AB^*^/^*^F^* are relatively lagging, reflecting a persistent disconnect between “local clarity” and “cross-modal information consistency.” DDcGAN and SwinFuse are obviously weak on *Q^AB^*^/^*^F^*, suggesting insufficient transfer of functional boundaries and anatomical edges; although FusionMamba is competitive on some metrics, combined with subsequent qualitative results, it is more prone to introducing noise and artifacts. SwinFusion and MambaDFuse are close to second-best on metrics such as MI, VIF, or *Q^AB^*^/^*^F^*, and are relatively balanced overall, but still struggle to simultaneously dominate in structural similarity, visual fidelity, and edge transfer.

On MRI–SPECT, MSMFusion shows its clearest gains on MI and PSNR. Its MI is 2.3275, above SwinFusion (2.2872), and its PSNR is 34.2085, above SAFusion (34.0290). MSMFusion is also highest on SSIM, VIF, and *Q^AB^*^/^*^F^*, although the differences are small: SSIM is 0.7993 against 0.7968 for SwinFusion, VIF is 1.0548 against 1.0493 for MambaDFuse, and *Q^AB^*^/^*^F^* is 0.7711 against 0.7630 for SwinFusion. Its EN occupies a middle position. At 3.6137, it exceeds SwinFusion (3.5239) and SAFusion (3.5441), while remaining below MMIFINet (5.3939) and FATFusion (4.7266). Its SF is 20.6452, lower than SwinFuse (20.7591), FusionMamba (22.3867), and MATR (23.6720). For MSMFusion, information retention and pixel agreement stand out, three further scores lead by narrow margins, and entropy together with spatial frequency do not lead.

### 4.3. Experimental Results and Discussions

#### 4.3.1. MRI–CT Experimental Results

**Figure 6:**
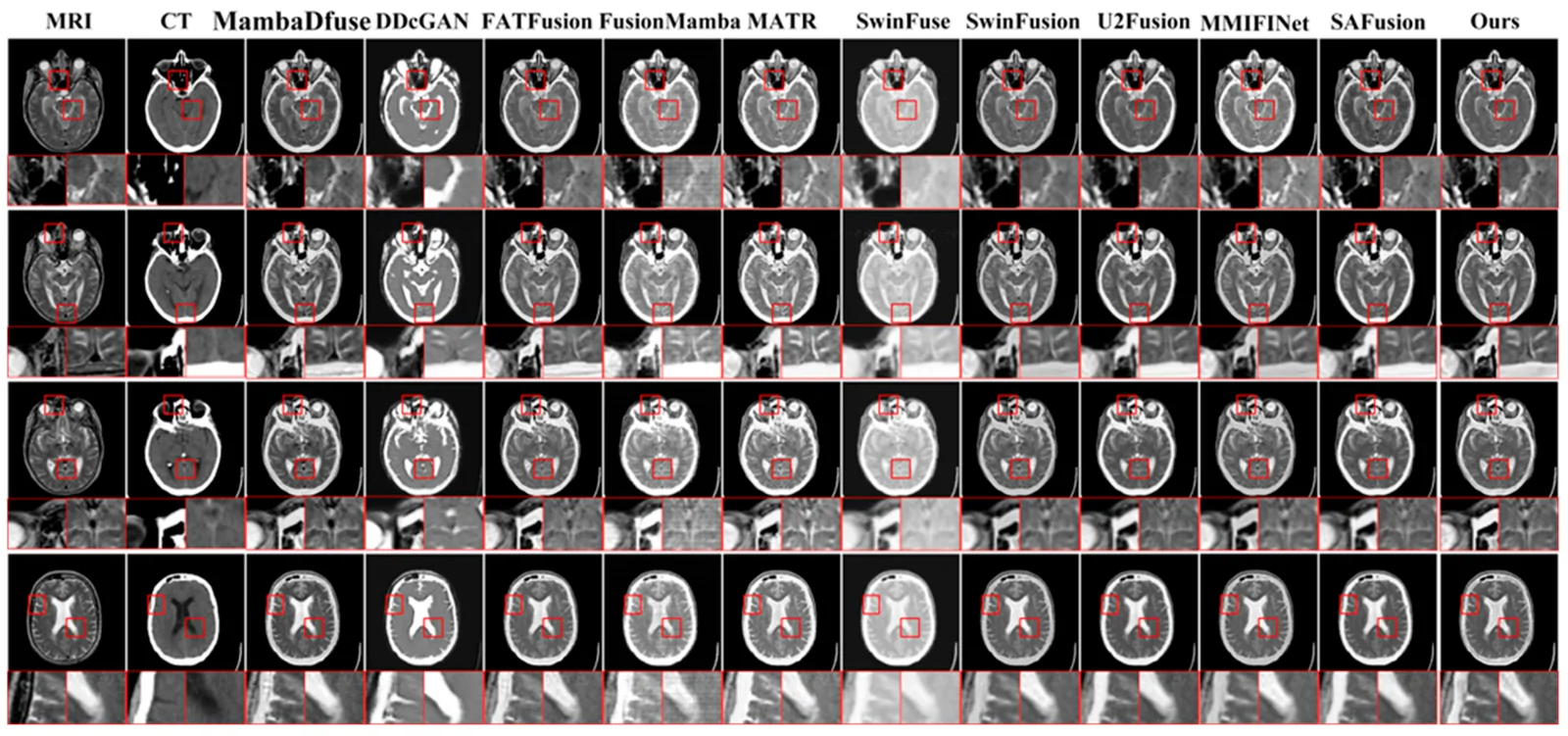
Qualitative results of four typical MRI and CT image fusion pairs. From left to right: MRI image, CT image, MambaDFuse fusion result, DDcGAN fusion result, FATFusion fusion result, FusionMamba fusion result, MATR fusion result, SwinFuse fusion result, SwinFusion fusion result, U2Fusion fusion result, and the proposed MSMFusion fusion result (ours).

Figure 6 shows four typical MRI–CT image pairs and the fusion results of different methods, with key local regions magnified for comparison. It can be seen that the results of DDcGAN, FusionMamba, MATR, SwinFuse, and SwinFusion are overall unsatisfactory in terms of luminance distribution and clarity; in particular, DDcGAN and SwinFuse are prone to artifacts and redundant textures, causing some details to be blurred. Although MambaDFuse, FATFusion, and U2Fusion can obtain relatively acceptable fusion appearances, they still show insufficient sharpening at bony edges and soft-tissue interfaces. In comparison, MSMFusion retains MRI texture and CT bone, with distinct boundaries and only moderate contrast. The boundaries agree with the higher SF, SSIM, and *Q^AB^*^/^*^F^* in Table 2. The moderate contrast agrees with the lower EN, which remains the disadvantage on this dataset.

#### 4.3.2. MRI–PET Experimental Results

**Figure 7:**
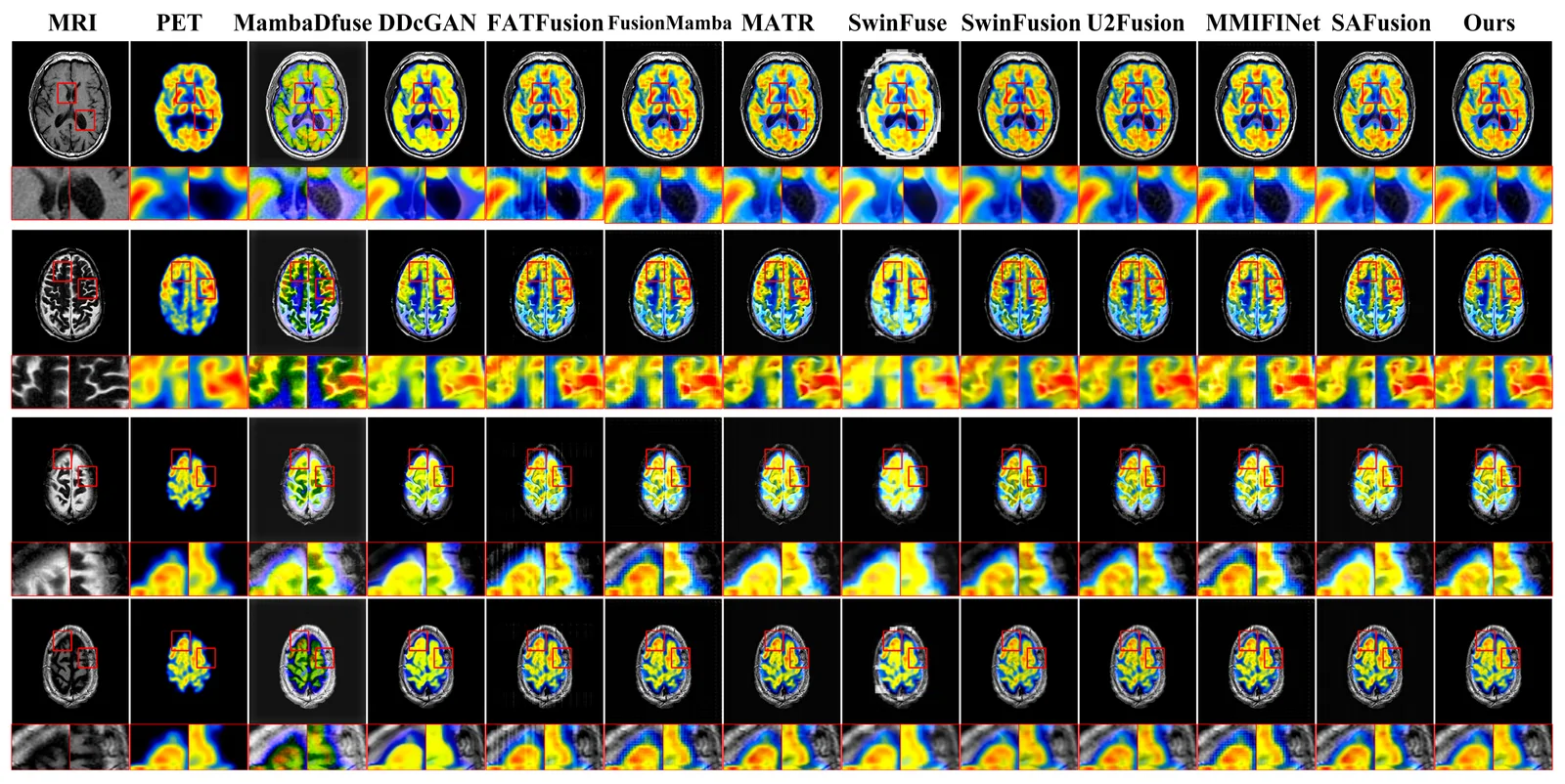
Qualitative results of four typical MRI and PET image fusion pairs. From left to right: MRI image, PET image, MambaDFuse fusion result, DDcGAN fusion result, FATFusion fusion result, FusionMamba fusion result, MATR fusion result, SwinFuse fusion result, SwinFusion fusion result, U2Fusion fusion result, and the proposed MSMFusion fusion result (ours).

Figure 7 presents the qualitative comparison of four typical MRI–PET fusion pairs. MambaDFuse shows insufficient detail preservation, with blocky artifacts visible in edge regions, and the local functional structures in PET are not sufficiently prominent in the fused image; DDcGAN produces blurred and discontinuous edges, with heavy loss of local details; FATFusion has difficulty accurately reflecting the structural features in PET; FusionMamba exhibits over-smoothing, leading to attenuation of high-frequency information; MATR has overall low contrast, and the fine MRI structures are not sufficiently clear; SwinFuse has insufficient edge sharpening accompanied by noise interference. SwinFusion and U2Fusion can achieve relatively satisfactory overall results, but still suffer from a certain degree of edge detail loss. In comparison, MSMFusion retains MRI texture, a uniform PET color, and aligned anatomical contours. These traits agree with the higher MI, VIF, edge preservation, PSNR, and SSIM, the last two by a small margin. FATFusion remains higher in EN, and FusionMamba and FATFusion remain higher in SF.

#### 4.3.3. MRI–SPECT Experimental Results

**Figure 8:**
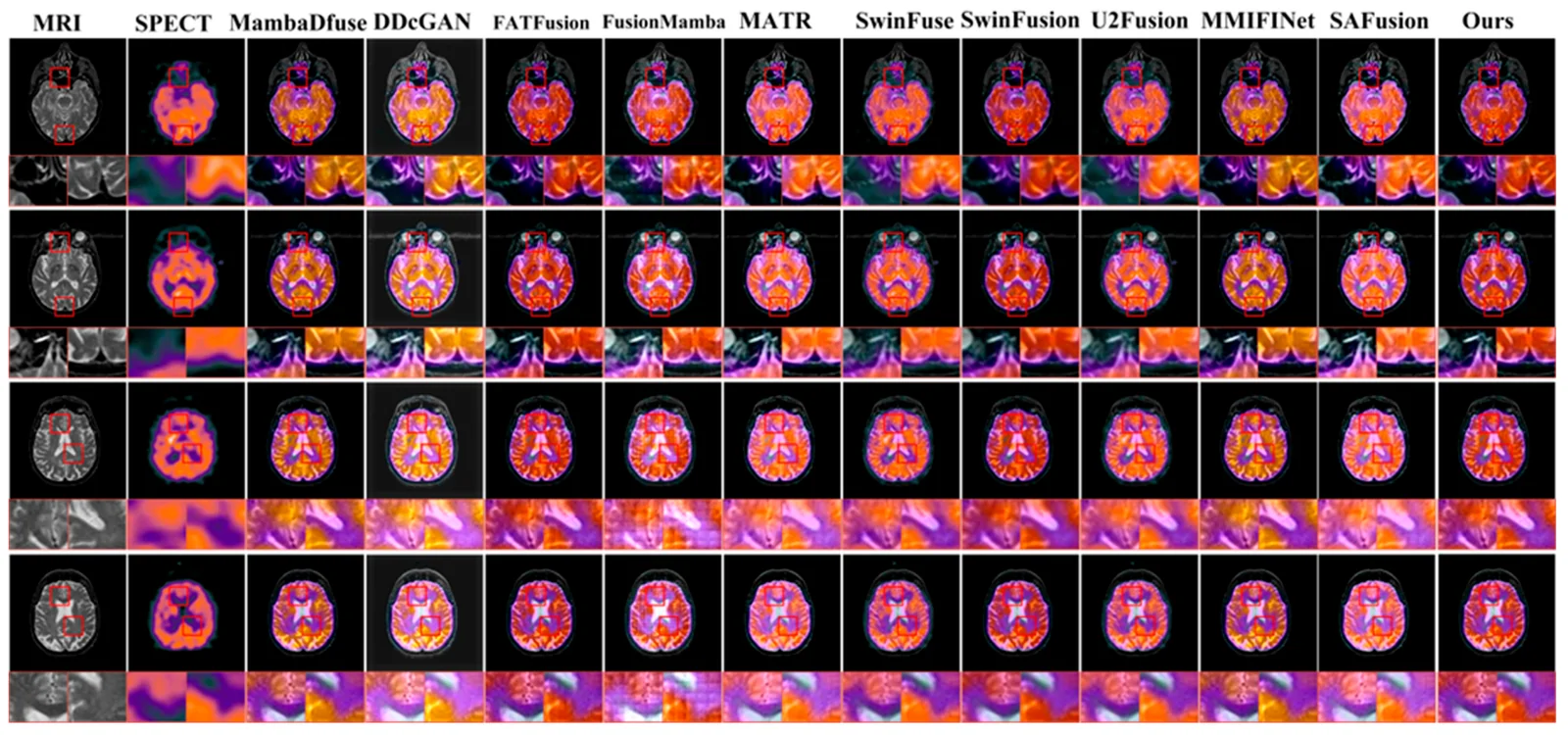
Qualitative results of four typical MRI and SPECT image fusion pairs. From left to right: MRI image, SPECT image, MambaDFuse fusion result, DDcGAN fusion result, FATFusion fusion result, FusionMamba fusion result, MATR fusion result, SwinFuse fusion result, SwinFusion fusion result, U2Fusion fusion result, and the proposed MSMFusion fusion result (ours).

Figure 8 shows four typical MRI–SPECT fusion results. Similar to the MRI–PET scenario, MSMFusion shows the SPECT color inside MRI boundaries. In comparison, noise and artifacts are visible in the fused images of FusionMamba; DDcGAN has weak color luminance and insufficient functional contrast; SwinFuse and U2Fusion exhibit over-smoothing with insufficient boundary clarity; although MambaDFuse, FATFusion, MATR, and SwinFusion can obtain relatively good overall appearances, they are still accompanied by varying degrees of detail loss. Clearer functional regions and MRI boundaries agree with the higher MI and PSNR. The slight gains in SSIM, VIF, and edge preservation are not visually separable from the nearest results. FATFusion remains higher in EN, and MATR remains higher in SF.

#### 4.3.4. Infrared–Visible Fusion on MSRS and RoadScene

MSMFusion is also applied to visible–infrared fusion, which tests its applicability outside brain imaging. MSRS and RoadScene each pair a visible image with an infrared image of a road scene, so both the modality combination and the scene differ from the Harvard MRI–CT, MRI–PET, and MRI–SPECT pairs. Table 5 and Table 6 report the same seven metrics. The values remain comparisons with the source images.

On the MSRS dataset (Table 5), EN, PSNR, and VIF are 6.6357, 30.7152, and 0.9082, each the highest. Edge preservation is 0.6295, above SwinFusion (0.6218). SSIM is 0.6766, also the highest, although the margin over MambaDFuse (0.6764) is small. MI is 2.5965, below MambaDFuse (2.7750). SF is 10.2671, below SAFusion (14.0400), FATFusion (12.6855), and FusionMamba (11.3138). Entropy, a disadvantage on all three Harvard tasks, is an advantage on this set. Spatial frequency is not.

On the RoadScene dataset (Table 6), MI, PSNR, and VIF are 2.4078, 29.2745, and 0.7910, each the highest. SSIM is 0.6311, also the highest, with a small margin over SwinFusion (0.6224). EN is 7.0404, below MambaDFuse (7.5311). Edge preservation is 0.5320, below MambaDFuse (0.5909). SF is 13.3370, below MambaDFuse (16.2776), SwinFusion (15.6431), MMIFINet (14.9387), and SAFusion (14.7503). Information retention, pixel agreement, structural similarity, and visual information are the advantages on this set. Entropy, edge transfer, and spatial frequency are not.

Across the two sets, PSNR and VIF are highest. Entropy is an advantage on MSRS and is not on RoadScene. Spatial frequency is lower on both.

### 4.4. Ablation Study

To evaluate the contribution of each core component in the MSMFusion framework, we conducted systematic ablation experiments on the MRI–CT, MRI–PET, and MRI–SPECT datasets, with the quantitative results summarized in Table 7, Table 8 and Table 9, respectively (bold denotes the best). Five metrics, namely MI, VIF, PSNR, SSIM, and *Q^AB^*^/^*^F^*, were adopted for quantitative assessment to validate the fusion results.

In Table 7, Table 8 and Table 9, a check mark means that the module is retained. An empty cell under MSFEM means that the multiscale extractor is replaced by a standard convolution. An empty cell under MSMamba means that the state-space blocks are removed. An empty cell under GAFM means that gated fusion is replaced by element-wise addition of the two modality features. The first three result rows change one module, and the next three rows change two modules. The last row is the complete model.

#### 4.4.1. Effectiveness of Core Components

Table 7, Table 8 and Table 9 correspond to the fusion tasks on the three different datasets, respectively, presenting the quantitative results of the network architectures designed under different ablation experiments. After replacing MSFEM with ordinary convolutions, the edge-related metrics decline most significantly. Taking MRI–CT as an example, *Q^AB^*^/^*^F^* decreases from 0.5876 of the full model to 0.5256, and MI decreases from 2.3280 to 2.0023; on MRI–PET, MI and *Q^AB^*^/^*^F^* decrease to 1.9015 and 0.6772, respectively. This indicates that, after lacking multiscale local representations, the model’s ability to capture fine-grained textures and boundary cues is weakened, which in turn affects cross-modal information retention and edge transfer. Removing MSMamba leads to an even larger performance degradation. On MRI–CT, MI, SSIM, and *Q^AB^*^/^*^F^* decrease to 0.8138, 0.5584, and 0.2952, respectively; on MRI–PET, SSIM even drops to 0.1974 and PSNR to 27.5253. Relying solely on local convolutional features makes it difficult to establish cross-modal long-range semantic associations and non-local context alignment; MSMamba is precisely the key support for maintaining structural consistency and global information interaction. After replacing GAFM with element-wise addition, the fusion quality also declines significantly. For example, on MRI–PET, SSIM and *Q^AB^*^/^*^F^* decrease to 0.1059 and 0.1442, respectively; on MRI–CT, SSIM and *Q^AB^*^/^*^F^* decrease to 0.5161 and 0.3777, respectively. Simple addition cannot adaptively assign the contributions of the two modalities according to spatial position, and easily causes contrast imbalance, diffusion of functional information, or submersion of anatomical details. On MI, VIF, PSNR, SSIM, and edge preservation, the full model is the highest row in Table 7, Table 8 and Table 9. Those tables do not include EN or SF, so they leave the entropy disadvantage, and the spatial-frequency disadvantage on MRI–PET and MRI–SPECT, unchanged.

On MRI–SPECT, replacing MSFEM reduces SSIM from 0.7690 to 0.6723 and the edge-preservation value from 0.7260 to 0.7079. Removing MSMamba reduces SSIM from 0.7690 to 0.3439 and PSNR from 33.8936 to 29.9963. Replacing GAFM with addition reduces SSIM from 0.7690 to 0.4736 and the edge-preservation value from 0.7260 to 0.5517. The full model is highest on MI, VIF, PSNR, SSIM, and edge preservation in this table.

#### 4.4.2. Effect of the Number of MSMamba Blocks

The influence of the number of stacked MSMamba blocks (1, 2, 3, 4) on fusion performance was validated on the MRI–PET dataset, and the results are shown in Table 10. With 3 blocks, MI, PSNR, SSIM, VIF, and SF are 2.08, 33.76, 0.77, 0.86, and 28.44, each the highest in Table 10. Edge preservation is 0.70, tied with 2 blocks. EN is 4.29, below 2 blocks (4.39) and 4 blocks (4.66); 3 blocks are kept for those five fidelity measures, not because entropy is highest.

When only 1 block is used, MI and PSNR are 1.96 and 32.69, respectively, significantly lower than the setting with 3 blocks, indicating that insufficient network depth limits the global context aggregation capability. After increasing to 2 blocks, some metrics recover, but SSIM drops to 0.47, suggesting that increasing depth does not always bring synchronous improvement in structural fidelity. When further increasing to 4 blocks, entropy exceeds the 3-block setting, but MI, PSNR, and SSIM decrease to 1.84, 31.06, and 0.43, respectively, exhibiting redundant modeling and even detail loss caused by excessively deep stacking. Therefore, 3 MSMamba blocks are adopted to achieve a more robust trade-off among representation capability, structural fidelity, and overfitting risk.

#### 4.4.3. Comparison with Transformer-Based Alternatives

**Figure 9:**
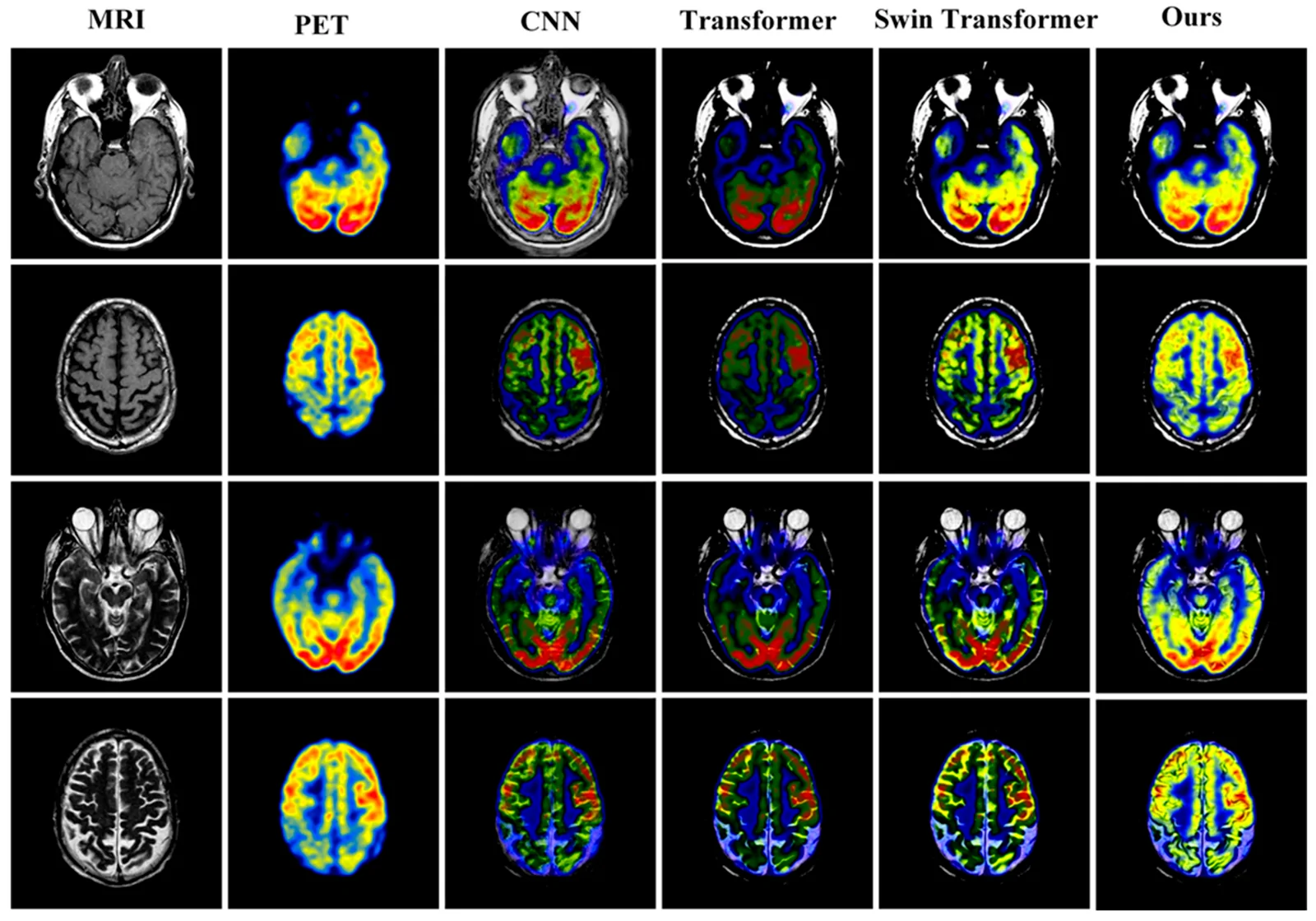
Qualitative comparison of fusion results among the Transformer, Swin Transformer, Conv, and MSMamba configurations on a representative MRI–PET image pair. It can be seen that the Transformer is prone to luminance attenuation and detail blurring; Swin Transformer shows some improvement but remains relatively soft; the Conv structure is clearer but the metabolic color distribution is not natural enough; MSMamba retains the PET functional information together with the fine anatomical details of MRI.

To further validate the effectiveness of MSMamba as the core global modeling unit, we replaced it with a standard Transformer or Swin Transformer while keeping MSFEM and GAFM unchanged, and conducted a qualitative comparison on MRI–PET (Figure 9). From the visual results, the Transformer variant is overall darker, with insufficient clarity of local boundaries and weak expression of functional information; although Swin Transformer shows some improvement, it still suffers from over-smoothing of details or insufficient contrast of functional pseudo-color in some regions. In comparison, MSMamba keeps the anatomical boundaries legible and the metabolic color stable relative to the two Transformer variants.

The computational complexity comparison under the same input size (1 × 256 × 256) is shown in Table 11. MSMamba (ours) has 4.154M parameters, 159.239G FLOPs, a model size of 15.86 MB, a single inference time of 58.92 ms, and an FPS of 16.97. In contrast, the Transformer solution increases the parameters and FLOPs to 7.201M and 476.738G, respectively, with an inference time of 94.11 ms and an FPS of only 10.63; the overhead of Swin Transformer is further increased (8.782M, 555.658G, 106.62 ms, and 9.38 FPS). This demonstrates that MSMamba keeps this result at a lower parameter count, computation, and latency than either Transformer variant.

**Table 11:** Computational complexity comparison of replacing MSMamba with Transformer modules.

Core Module	Params (M)	FLOPs (G)	Size (MB)	Time (ms)	FPS
Transformer	7.201	476.738	27.47	94.11	10.63
Swin Transformer	8.782	555.658	33.69	106.62	9.38
MSMamba (Ours)	**4.154**	**159.239**	**15.86**	**58.92**	**16.97**

#### 4.4.4. Ablation on Scanning-Path Designs

To verify the necessity of the dual-path scanning expansion strategy in MSMamba, we ablated the scanning path configuration on the MRI–PET dataset, and the results are shown in Table 12. The specific settings are as follows: V1 employs only two full-resolution scanning paths; V2 retains only the downsampled scanning paths; V3 adopts a four-branch full-resolution scan without downsampling; V4 is the complete MSMFusion method proposed in this paper, i.e., a combination of two full-resolution paths and two downsampled paths. In addition to fusion quality metrics such as MI, PSNR, SSIM, VIF, and *Q^AB^*^/^*^F^*, Table 12 also reports the number of paths (Paths), the relative scanning sequence volume (Rel. Tokens), and the inference time (Time) to characterize the computational overhead of different scanning strategies.

For ease of comparison, let the full-resolution sequence length be *L* = *HW*, and the sequence length of the stride-2 downsampled path be L/4. The total scanning sequence volume of each variant is obtained by summing the sequence lengths of all paths, and is normalized by V1 to obtain Rel. Tokens. Accordingly, the absolute sequence volumes of V1–V4 are 2 L, 0.5 L, 4 L and 2.5 L, corresponding to Rel. Tokens of 1.00, 0.25, 2.00, and 1.25, respectively.

The quantitative results clearly reveal that “details only,” “long-range only,” or “blindly increasing same-scale scanning” all fail to achieve the optimal trade-off. First, although V1 retains full-resolution detail scanning, its SSIM reaches only 0.2513 and PSNR drops to 27.6213, indicating that without the downsampled long-range path, the model struggles to establish sufficient global context associations, and structural fidelity is significantly impaired. Second, V2 relies solely on the downsampled paths, with the lowest inference time (41.77 ms) and Rel. Tokens of only 0.25, making it the most lightweight in computation; however, its MI drops to 1.8626, indicating that fine-grained texture and boundary localization information is excessively weakened in the compressed sequences, resulting in insufficient cross-modal information retention. Third, V3 places all four paths at full resolution, with Rel. Tokens rising to 2.00 and the highest inference time (61.59 ms); nevertheless, it is lower than V4 on the quality metrics, including VIF at 0.7566 and *Q^AB^*^/^*^F^* of 0.6587. This demonstrates that additional same-scale full-resolution scans mainly introduce redundant computation and feature duplication, and cannot be stably translated into higher fusion gains.

V4 is highest on each of the five quality metrics. MI, PSNR, SSIM, VIF, and *Q^AB^*^/^*^F^* are 2.2181, 32.9390, 0.7616, 0.8208, and 0.6965, respectively. Compared with V3, V4 reduces the scanning sequence from 4 L to 2.5 L (Rel. Tokens from 2.00 to 1.25) and shortens the inference time. It is not the fastest variant: 59.31 ms, against 41.77 ms for V2 and 47.40 ms for V1. These results indicate that the full-resolution branch is responsible for retaining the fine-grained cues required for boundaries and textures, while the downsampled branch completes efficient long-range modeling on shorter sequences; the complementarity of the two can improve fusion quality while reducing scanning redundancy. In other words, the advantage of MSMamba does not come from more scanning paths, but from a reasonable combination of resolution-heterogeneous paths, thereby achieving a better balance among detail fidelity, global dependencies, and computational overhead.

**Table 12:** Ablation on MSMamba scanning-path designs on the MRI–PET dataset.

Variant	Paths	Rel. Tokens	Time (ms)	MI	PSNR	SSIM	VIF	*Q^AB^* ^/^ *^F^*
V1: Single-path MSMamba	2	1.00	47.40	2.0337	27.6213	0.2513	0.6665	0.6711
V2: Downsampled-path only	2	0.25	41.77	1.8626	32.2897	0.7405	0.8175	0.6903
V3: Dual-path w/o Downsampling	4	2.00	61.89	2.0781	32.1898	0.7527	0.7566	0.6587
V4: Proposed MSMamba	4	1.25	59.31	2.2181	32.9390	0.7616	0.8208	0.6965

## 5. Parameter Analysis

To analyze how the loss weights affect fusion performance, as shown in Table 13, each weight was varied on the MRI–PET task while the others were held at the selected values. λ1 weights the intensity loss, λ2 weights the gradient loss, λ3 weights the structural similarity loss, and λ4 weights the total-variation loss. The selected setting is λ1 = 20, λ2 = 100, λ3 = 100, and λ4 = 1. Around this setting, λ1 was set to 5 and to 80, λ2 was set to 25 and to 400, λ3 was set to 25 and to 400, and λ4 was set to 0 and to 10. A further setting assigned the value 1 to all four weights. Table 13 reports EN, SF, MI, PSNR, SSIM, VIF, and the edge-preservation score for these combinations.

Moving any one weight away from the selected setting lowers the metrics associated with that term. With λ2, λ3, and λ4 fixed, λ1 = 5 lowers VIF from 0.82 to 0.44 and SSIM from 0.76 to 0.49, and λ1 = 80 lowers them to 0.64 and 0.55. Setting all four weights to 1 lowers MI from 2.22 to 1.75. With the remaining weights fixed, λ2 = 25 lowers VIF to 0.42, and λ2 = 400 lowers SSIM to 0.30 and PSNR to 29.16. λ3 = 25 lowers SSIM to 0.33 and PSNR to 29.60, and λ3 = 400 lowers SSIM to 0.45. Removing the total-variation term lowers VIF to 0.51, and raising λ4 to 10 lowers the edge-preservation score to 0.43. At λ1 = 20, λ2 = 100, λ3 = 100, and λ4 = 1, SF, MI, PSNR, SSIM, VIF, and the edge-preservation score are the highest values in Table 13: 25.35, 2.22, 32.94, 0.76, 0.82, and 0.70. EN is 4.34, below the 4.39 obtained when λ1 = 5. These weights are kept because of the six higher scores.

## 6. Limitations and Future Work

Although MSMFusion achieves excellent performance and computational efficiency in structural–functional medical image fusion, it is still necessary to objectively recognize the conditions that constrain its generalization capability. The present model fuses registered two-dimensional slices. Lipid distributions in three-dimensional pancreatic-cancer spheroids change through the volume rather than within one section [58]. Slice-wise fusion therefore leaves this through-plane information unrepresented. Similar to most existing deep learning fusion models, MSMFusion strongly relies on perfect spatial registration of the input source images; for example, the SS2D scanning in MSMamba depends on the spatial continuity of images, and if the source images exhibit slight misalignment or local deformation, the scanning paths will introduce erroneous spatial context, while GAFM can only adjust the weights of already aligned features and cannot correct geometric deviations.

In response to the above limitations, we plan to conduct in-depth exploration in the following aspects in future work. First, we will extend to high-dimensional medical data processing, exploring the upgrade of the existing multiscale feature extraction and MSMamba modules to three-dimensional architectures (e.g., 3D-Mamba), so as to directly process 3D or even 4D spatiotemporal medical data and thereby more comprehensively capture long-range dependencies within volumetric space. Three-dimensional medical image fusion already combines a convolutional encoder with state-space modeling [42]. Extending the present MSMamba to volumes is constrained by the memory overhead of three-dimensional state-space scanning. For a volume of size H×W×D, each scanning direction is serialized into HWD tokens. During training, selective scanning stores hidden states along the sequence, so the activation memory scales with the voxel count and the number of scan directions. MSMamba already keeps two paths at full resolution. Placing those paths on the native voxel grid and adding directions along the slice axis yields much longer sequences than the current two-dimensional scan, in which a stride-2 path reduces the sequence length to one quarter. The feasible implementation is to retain full-resolution scanning within each axial slice, to represent through-plane context on a downsampled grid, and to checkpoint the scan states during backpropagation, rather than expanding every scanning direction over the full voxel lattice. Second, to enhance the model’s adaptability to complex clinical data, we plan to integrate feature-level deformable registration or cross-attention alignment mechanisms into the fusion framework, enabling the network to adaptively perform alignment and fusion on source images with slight spatial deviations, thereby significantly improving the robustness and generalization capability of the model. Third, to lower the cost of possible future use, we will also focus on lightweight model design, exploring strategies such as structural re-parameterization, knowledge distillation, low-rank approximation, or efficient Mamba variants, with the aim of substantially reducing the number of model parameters and inference time while maintaining fusion accuracy, and lowering computational resource overhead.

## 7. Conclusions

To address the problems of insufficient local detail preservation, high cost of global dependency modeling, and difficulty in adaptively integrating cross-modal information in multimodal medical image fusion, we propose a multiscale Mamba-based fusion framework, MSMFusion. The method is centered on the collaboration between CNNs and state space models: first, a multiscale feature extraction module captures modality-specific multiscale local structures; subsequently, a multiscale Mamba module establishes long-range spatial dependencies at linear complexity, and adopts a dual-path scanning strategy combining full-resolution and downsampled paths to balance fine-grained details and efficient global modeling; finally, a gated adaptive fusion module selectively weights the bimodal features according to spatial content, generating high-quality fused images that simultaneously preserve anatomical structures and functional information.

Extensive experiments on the public Harvard medical imaging dataset demonstrate that MSMFusion achieves competitive fusion performance on all three tasks of MRI–CT, MRI–PET, and MRI–SPECT. Compared with various representative methods, our method is highest on MI, VIF, and *Q^AB^*^/^*^F^*. PSNR and SSIM are highest as well, with a substantial PSNR margin on MRI–SPECT and a slight margin on MRI–PET. EN is lower on all three tasks. SF is highest only on MRI–CT and is lower on MRI–PET and MRI–SPECT. Fidelity and information retention are the advantages; entropy is the disadvantage on every task, and spatial frequency is the disadvantage on the two functional tasks.

The same model is also applicable to visible–infrared fusion on MSRS and RoadScene. PSNR and VIF are highest on both sets. Entropy is highest on MSRS and is not on RoadScene. Spatial frequency is lower on both.

These results are confined to two-dimensional slices. MSMFusion does not model continuity along the *Z*-axis, so cross-slice spatial context is absent from the fused image. This is a significant limitation for clinical image fusion: performance on registered slice pairs does not establish generalizability to volumetric clinical use. The metric comparisons describe fusion quality on public images. Where the scores are higher, they indicate better preservation of structural and functional information. These metric results aid clinical interpretation.

## Figures and Tables

**Table 1:** Comparison of Mamba-based image fusion methods in terms of scanning strategy, multiscale modeling, and cross-modal fusion.

Method	Scanning Strategy	Multiscale Modeling	Adaptive Fusion Design
VMamba [36]	Four directional scans, all at full resolution.	Hierarchical backbone with stage-wise patch merging. Each SS2D scan stays at that stage’s resolution. No fusion head.	No cross-modal fusion.
MambaDFuse [39]	Selective scan on a single-resolution feature sequence.	Dual-level stack: shallow convolution and deep Mamba, without parallel dilated fields.	Fixed channel exchange at the shallow stage; modality-guided M3 block at the deep stage.
FusionMamba [40]	Efficient 2D scan: every path is skip-sampled, and a local convolution repairs neighborhood loss.	U-Net hierarchy by patch embedding and stage-wise patch merging.	Dynamic texture and difference enhancement, then a cross-modal Mamba module. Fusion is inside the scan.
SSEFusion [41]	Mamba long-range modeling. Scan resolution is not the design focus.	Salient semantic enhancement, rather than a dilated multi-receptive-field front-end.	Mamba coupled with dynamic spiking neural networks, oriented to salient semantics.
MSMFusion (Ours)	Dual path: two full-resolution scans and two stride-2 scans, merged after upsampling.	MSFEM before scanning: dilated rates 6, 12, and 18, plus global pooling and channel–spatial attention.	GAFM after independent modality modeling: a learned spatial–channel gate in (0, 1).

**Table 2:** Quantitative comparison results of MRI–CT fusion. Bold and underline denote the best and second-best values, respectively.

Method	EN	SF	MI	PSNR	SSIM	VIF	*Q^AB^* ^/^ *^F^*
MambaDFuse	5.4149 ± 0.1812	30.0409 ± 3.7806	2.1368 ± 0.0973	30.0275 ± 0.1325	0.3704 ± 0.0109	0.5983 ± 0.0435	0.5285 ± 0.0428
DDcGAN	4.5653 ± 0.2758	24.4711 ± 1.9353	2.0106 ± 0.0931	27.5431 ± 1.1509	0.1983 ± 0.0089	0.4495 ± 0.0330	0.2249 ± 0.0100
FATFusion	4.9414 ± 0.1227	25.2101 ± 1.9773	2.1348 ± 0.0916	31.0700 ± 0.2432	0.7115 ± 0.0157	0.6792 ± 0.0283	0.2252 ± 0.0216
FusionMamba	4.2171 ± 0.1308	31.6786 ± 4.6527	2.0365 ± 0.0876	29.5728 ± 0.0984	0.3176 ± 0.0101	0.5601 ± 0.0262	0.4063 ± 0.0412
MATR	5.3666 ± 0.1587	25.1117 ± 1.5801	2.0028 ± 0.0992	30.0792 ± 0.2471	0.6919 ± 0.0110	0.5371 ± 0.0267	0.2655 ± 0.0152
SwinFuse	4.2084 ± 0.2030	10.3489 ± 1.5491	2.1535 ± 0.0952	27.1012 ± 0.0709	0.6728 ± 0.0120	0.4949 ± 0.0266	0.1273 ± 0.0161
SwinFusion	4.9518 ± 0.1792	29.5727 ± 3.4595	2.2353 ± 0.1179	31.4927 ± 0.1856	0.7038 ± 0.0203	0.6984 ± 0.0318	0.5635 ± 0.0371
U2Fusion	4.6175 ± 0.1853	20.7834 ± 2.4400	2.2613 ± 0.1104	32.0592 ± 0.3360	0.6836 ± 0.0233	0.7022 ± 0.0460	0.5129 ± 0.0277
MMIFINet	**5.5939 ± 0.0820**	28.2593 ± 2.7950	1.6791 ± 0.1036	30.2182 ± 0.1846	0.6323 ± 0.0156	0.4937 ± 0.0281	0.4982 ± 0.0124
SAFusion	4.5068 ± 0.1892	29.3310 ± 4.5938	2.1549 ± 0.1233	31.7500 ± 0.3534	0.7180 ± 0.0192	0.6774 ± 0.0362	0.5446 ± 0.0270
Ours	4.9133 ± 0.2715	**32.0921 ± 4.5360**	**2.3306 ± 0.1144**	**32.3425 ± 0.1832**	**0.7267 ± 0.0202**	**0.7166 ± 0.0285**	**0.5876 ± 0.0423**

**Table 3:** Quantitative comparison results of MRI–PET fusion. Bold and underline denote the best and second-best values, respectively.

Method	EN	SF	MI	PSNR	SSIM	VIF	*Q^AB^* ^/^ *^F^*
MambaDFuse	4.2328 ± 0.6822	24.9073 ± 4.7548	2.1507 ± 0.2832	31.1529 ± 1.1231	0.7565 ± 0.0465	0.7895 ± 0.0560	0.6870 ± 0.0681
DDcGAN	3.8174 ± 0.5583	22.4081 ± 2.3615	1.7712 ± 0.2046	32.2568 ± 1.0142	0.6848 ± 0.0604	0.5365 ± 0.0634	0.6494 ± 0.0612
FATFusion	5.0443 ± 0.3378	25.6874 ± 1.9543	1.7608 ± 0.2524	28.4030 ± 0.1754	0.6617 ± 0.0406	0.6613 ± 0.1310	0.6366 ± 0.0648
FusionMamba	4.3212 ± 0.3754	**26.9397 ± 5.1547**	1.8778 ± 0.2471	30.2029 ± 0.2791	0.3104 ± 0.0389	0.7325 ± 0.1547	0.5893 ± 0.1132
MATR	4.1435 ± 0.6167	22.9843 ± 2.3941	1.7323 ± 0.2236	30.4936 ± 0.3756	0.6623 ± 0.0435	0.5700 ± 0.1084	0.3120 ± 0.0407
SwinFuse	4.1941 ± 0.7354	21.6367 ± 2.7936	1.7249 ± 0.2189	31.2066 ± 0.9867	0.6352 ± 0.0346	0.4616 ± 0.0540	0.6538 ± 0.0313
SwinFusion	4.2530 ± 0.7137	25.3063 ± 5.0661	2.1703 ± 0.2559	32.9349 ± 1.0733	0.7513 ± 0.0460	0.8140 ± 0.1689	0.6798 ± 0.0880
U2Fusion	4.3120 ± 0.7538	17.7674 ± 3.1474	2.0766 ± 0.2571	32.5941 ± 1.1970	0.7607 ± 0.0481	0.7822 ± 0.1465	0.6121 ± 0.0959
MMIFINet	**5.5390 ± 0.4304**	24.5139 ± 5.3978	1.6102 ± 0.1649	29.9211 ± 0.2630	0.6086 ± 0.0487	0.5588 ± 0.0904	0.6183 ± 0.0790
SAFusion	3.9455 ± 0.6585	26.6431 ± 5.8866	2.1834 ± 0.2809	31.7926 ± 1.2296	0.6630 ± 0.0468	0.7915 ± 0.1826	0.6845 ± 0.0829
Ours	4.3326 ± 0.7086	25.3561 ± 5.1207	**2.2181 ± 0.2653**	**32.9390 ± 1.1838**	**0.7616 ± 0.0461**	**0.8208 ± 0.1633**	**0.6965 ± 0.0886**

**Table 4:** Quantitative comparison results of MRI–SPECT fusion. Bold and underline denote the best and second-best values, respectively.

Method	EN	SF	MI	PSNR	SSIM	VIF	*Q^AB^* ^/^ *^F^*
MambaDFuse	3.9907 ± 0.3721	20.3076 ± 4.3288	2.0333 ± 0.2003	33.5224 ± 0.4974	0.7670 ± 0.0360	1.0493 ± 0.0286	0.7522 ± 0.0407
DDcGAN	4.1716 ± 0.4788	19.7329 ± 0.8772	1.6702 ± 0.2241	32.0556 ± 0.5479	0.6933 ± 0.0376	0.6298 ± 0.0458	0.1903 ± 0.0507
FATFusion	4.7266 ± 0.2225	15.8501 ± 2.3507	1.9476 ± 0.1845	31.9542 ± 0.5404	0.7530 ± 0.0282	0.7469 ± 0.0529	0.4401 ± 0.0233
FusionMamba	4.5823 ± 0.3070	22.3867 ± 4.1154	1.6811 ± 0.1848	31.0230 ± 0.5294	0.4219 ± 0.0214	0.6927 ± 0.0816	0.5475 ± 0.0526
MATR	4.2764 ± 0.2930	**23.6720 ± 2.0518**	1.7305 ± 0.1771	28.8042 ± 0.2985	0.6458 ± 0.0253	0.6805 ± 0.0389	0.4206 ± 0.0618
SwinFuse	4.0084 ± 0.4954	20.7591 ± 1.1319	1.8710 ± 0.2036	25.4300 ± 0.2216	0.7417 ± 0.0263	0.5135 ± 0.0561	0.2326 ± 0.0646
SwinFusion	3.5239 ± 0.3633	20.3701 ± 4.5711	2.2872 ± 0.2369	32.0937 ± 0.5520	0.7968 ± 0.0357	1.0038 ± 0.0244	0.7630 ± 0.0392
U2Fusion	3.8540 ± 0.4348	19.9104 ± 2.3962	1.9223 ± 0.1882	33.3023 ± 0.8329	0.7068 ± 0.0376	0.8791 ± 0.0773	0.6511 ± 0.0699
MMIFINet	**5.3939 ± 0.2708**	19.2590 ± 3.8773	1.4101 ± 0.1323	29.5633 ± 0.1029	0.4878 ± 0.0245	0.6065 ± 0.0402	0.6779 ± 0.0453
SAFusion	3.5441 ± 0.4319	20.0691 ± 4.5097	2.2810 ± 0.2227	34.0290 ± 1.0040	0.7801 ± 0.0361	0.9835 ± 0.1051	0.7519 ± 0.0554
Ours	3.6137 ± 0.3509	20.6452 ± 4.7284	**2.3275 ± 0.2153**	**34.2085 ± 0.6736**	**0.7993 ± 0.0362**	**1.0548 ± 0.0317**	**0.7711 ± 0.0410**

**Table 5:** Quantitative comparison results of infrared–visible fusion on the MSRS dataset. Bold and underline denote the best and second-best values, respectively.

Method	EN	SF	MI	PSNR	SSIM	VIF	*Q^AB^* ^/^ *^F^*
MambaDFuse	5.4745 ± 0.5498	5.7450 ± 1.8440	**2.7750 ± 0.2861**	29.1494 ± 0.8217	0.6764 ± 0.0711	0.7397 ± 0.0759	0.3429 ± 0.1327
DDcGAN	5.2218 ± 0.2880	10.3326 ± 1.3315	1.2010 ± 0.1974	28.3893 ± 0.5153	0.2839 ± 0.0624	0.7566 ± 0.1758	0.1084 ± 0.0271
FATFusion	6.0592 ± 0.5600	12.6855 ± 1.2003	1.1774 ± 0.4264	28.1014 ± 1.2448	0.2692 ± 0.0811	0.5753 ± 0.0954	0.2748 ± 0.0402
FusionMamba	6.5448 ± 0.6855	11.3138 ± 3.3569	1.2025 ± 0.3953	28.8413 ± 0.6817	0.5234 ± 0.0490	0.7219 ± 0.1253	0.3161 ± 0.0524
MATR	5.4719 ± 0.5624	10.3002 ± 2.6083	1.3219 ± 0.2668	29.3646 ± 1.2751	0.2996 ± 0.0893	0.5015 ± 0.0852	0.2530 ± 0.0390
SwinFuse	5.6030 ± 0.7713	9.9054 ± 1.9832	1.8077 ± 0.4092	29.2399 ± 1.3364	0.2854 ± 0.0862	0.4251 ± 0.0837	0.1578 ± 0.0538
SwinFusion	6.5724 ± 0.7434	11.0815 ± 3.4267	2.2940 ± 0.3657	30.5926 ± 1.0644	0.6285 ± 0.0832	0.8637 ± 0.0873	0.6218 ± 0.0525
U2Fusion	6.5819 ± 0.6290	9.0917 ± 2.6202	2.2094 ± 0.3141	30.0265 ± 1.3160	0.6470 ± 0.0788	0.8453 ± 0.0742	0.5960 ± 0.0468
MMIFINet	5.7213 ± 0.3184	9.2467 ± 2.6322	0.8090 ± 0.2034	28.1063 ± 0.2936	0.6181 ± 0.0658	0.6015 ± 0.1002	0.5095 ± 0.0465
SAFusion	6.4328 ± 0.7348	**14.0400 ± 4.1652**	2.0264 ± 0.2946	30.1388 ± 1.3845	0.5841 ± 0.1238	0.8460 ± 0.1290	0.5646 ± 0.0497
Ours	**6.6357 ± 0.7745**	10.2671 ± 3.3499	2.5965 ± 0.6756	**30.7152 ± 0.9711**	**0.6766 ± 0.0704**	**0.9082 ± 0.1128**	**0.6295 ± 0.0828**

**Table 6:** Quantitative comparison results of infrared–visible fusion on the RoadScene dataset. Bold and underline denote the best and second-best values, respectively.

Method	EN	SF	MI	PSNR	SSIM	VIF	*Q^AB^* ^/^ *^F^*
MambaDFuse	**7.5311 ± 0.2917**	**16.2776 ± 3.6853**	2.2107 ± 0.4036	28.9554 ± 0.6351	0.6138 ± 0.0614	0.7656 ± 0.0752	**0.5909 ± 0.0692**
DDcGAN	5.6509 ± 0.2164	13.0297 ± 3.2039	1.7362 ± 0.2408	27.8997 ± 0.1949	0.2170 ± 0.0327	0.5037 ± 0.0828	0.1789 ± 0.0335
FATFusion	6.0778 ± 0.3250	11.7148 ± 1.7883	1.5396 ± 0.3032	27.8812 ± 0.1624	0.5337 ± 0.0456	0.5432 ± 0.0899	0.2450 ± 0.0303
FusionMamba	6.5687 ± 0.7112	11.3670 ± 5.4393	1.5780 ± 0.3490	28.2637 ± 0.2994	0.5749 ± 0.0452	0.6930 ± 0.1291	0.3679 ± 0.0525
MATR	6.1892 ± 0.2774	11.8647 ± 2.1229	1.7627 ± 0.3909	27.7397 ± 0.1610	0.5828 ± 0.0646	0.4972 ± 0.1105	0.2685 ± 0.0309
SwinFuse	5.4004 ± 0.6723	5.8997 ± 1.4032	1.8740 ± 0.3759	27.9053 ± 0.1574	0.5146 ± 0.0782	0.3993 ± 0.1182	0.1785 ± 0.0424
SwinFusion	6.9398 ± 0.3110	15.6431 ± 3.6153	1.7271 ± 0.4359	28.3936 ± 0.3284	0.6224 ± 0.0603	0.6521 ± 0.1554	0.5258 ± 0.0457
U2Fusion	6.9982 ± 0.2714	11.6046 ± 2.7680	2.0579 ± 0.4572	28.7836 ± 0.4522	0.5904 ± 0.0628	0.7453 ± 0.1504	0.5287 ± 0.0656
MMIFINet	6.6539 ± 0.2877	14.9387 ± 4.1214	1.0377 ± 0.2457	27.8011 ± 0.1525	0.5761 ± 0.0525	0.5977 ± 0.0874	0.5192 ± 0.0603
SAFusion	7.0163 ± 0.2054	14.7503 ± 5.7048	2.2304 ± 0.5755	28.6327 ± 0.5843	0.5750 ± 0.0774	0.7514 ± 0.1825	0.5165 ± 0.0588
Ours	7.0404 ± 0.2831	13.3370 ± 3.5350	**2.4078 ± 0.3485**	**29.2745 ± 0.2845**	**0.6311 ± 0.0941**	**0.7910 ± 0.1398**	0.5320 ± 0.0831

**Table 7:** Ablation study results on the MRI–CT dataset.

MSFEM	MSMamba	GAFM	MI	VIF	PSNR	SSIM	*Q^AB^* ^/^ *^F^*
	√	√	2.0023	0.6747	31.2146	0.6953	0.5256
√		√	0.8138	0.4464	31.3176	0.5584	0.2952
√	√		1.9878	0.6692	31.0441	0.5161	0.3777
√			2.1170	0.5734	31.1277	0.6662	0.4770
	√		2.1044	0.5824	26.8697	0.2607	0.4225
		√	1.9392	0.6775	30.4995	0.3488	0.5233
√	√	√	**2.3280**	**0.7177**	**32.4088**	**0.7321**	**0.5876**

**Table 8:** Ablation study results on the MRI–PET dataset.

MSFEM	MSMamba	GAFM	MI	VIF	PSNR	SSIM	*Q^AB^* ^/^ *^F^*
	√	√	1.9015	0.8049	32.8281	0.6996	0.6772
√		√	1.7228	0.6538	27.5253	0.1974	0.4915
√	√		1.3635	0.4431	28.3251	0.1059	0.1442
√			1.4382	0.5412	28.3509	0.2577	0.4119
	√		1.9045	0.8085	27.8309	0.2703	0.6655
		√	2.0093	0.6746	30.4995	0.3488	0.5233
√	√	√	**2.0674**	**0.8211**	**33.1240**	**0.7627**	**0.6941**

**Table 9:** Ablation study results on the MRI–SPECT dataset.

MSFEM	MSMamba	GAFM	MI	VIF	PSNR	SSIM	*Q^AB^* ^/^ *^F^*
	√	√	2.0381	0.8293	32.0811	0.6723	0.7079
√		√	1.9520	0.7874	29.9963	0.3439	0.6737
√	√		2.0049	0.6632	30.3545	0.4736	0.5517
√			1.3799	0.5050	31.4525	0.6542	0.4845
	√		1.6586	0.7354	30.8764	0.6284	0.5535
		√	1.9614	0.8659	31.1884	0.3087	0.6761
√	√	√	**2.2347**	**0.9622**	**33.8936**	**0.7690**	**0.7260**

**Table 10:** Effect of the number of MSMamba blocks on the MRI–PET dataset.

Blocks	EN	SF	MI	PSNR	SSIM	VIF	*Q^AB^* ^/^ *^F^*
1	4.03	27.57	1.96	32.69	0.66	0.82	0.69
2	4.39	28.07	1.97	32.74	0.47	0.83	0.70
4	**4.66**	27.81	1.84	31.06	0.43	0.78	0.68
3 (Ours)	4.29	**28.44**	**2.08**	**33.76**	**0.77**	**0.86**	**0.70**

**Table 13:** Quantitative evaluation results of the MRI–PET dataset under different parameter combinations. Bold font indicates the best results.

λ1, λ2, λ3, λ4	EN	SF	MI	PSNR	SSIM	VIF	*Q^AB^* ^/^ *^F^*
1, 1, 1, 1	4.03	25.31	1.75	31.41	0.63	0.79	0.68
5, 100, 100, 1	**4.39**	19.31	2.14	31.15	0.49	0.44	0.69
80, 100, 100, 1	4.11	22.20	2.21	31.23	0.55	0.64	0.59
20, 25, 100, 1	4.29	18.33	1.98	32.14	0.57	0.42	0.69
20, 400, 100, 1	3.15	23.72	2.03	29.16	0.30	0.72	0.63
20, 100, 25, 1	4.12	20.84	2.11	29.60	0.33	0.78	0.68
20, 100, 400, 1	3.78	17.60	2.19	31.66	0.45	0.70	0.57
20, 100, 100, 0	4.03	23.01	1.86	30.92	0.44	0.51	0.68
20, 100, 100, 10	4.32	24.00	2.12	31.13	0.49	0.80	0.43
20, 100, 100, 1 (Ours)	4.34	**25.35**	**2.22**	**32.94**	**0.76**	**0.82**	**0.70**

## Data Availability

The medical image data used in this study are available from the Harvard Whole Brain Atlas (https://www.med.harvard.edu/AANLIB/, accessed on 17 August 2026). The MSRS infrared–visible images are available at https://github.com/Linfeng-Tang/MSRS(accessed on 23 September 2026), and the RoadScene infrared–visible images are available at https://github.com/hanna-xu/RoadScene(accessed on 23 September 2026).
